# Discovery of a novel natural killer cell line with distinct immunostimulatory and proliferative potential as an alternative platform for cancer immunotherapy

**DOI:** 10.1186/s40425-019-0612-2

**Published:** 2019-05-24

**Authors:** Hyun Gul Yang, Moon Cheol Kang, Tae Yoon Kim, Injung Hwang, Hyun Tak Jin, Young Chul Sung, Ki-Seong Eom, Sae Won Kim

**Affiliations:** 1 Division of Integrative Biosciences and Biotechnology, Pohang University of Science and Technology (POSTECH), 77 Cheongam-Ro, Nam-Gu, Pohang, Gyeongbuk 37673 Republic of Korea; 2 SL-BIGEN Inc., 700 Daewangpanyo-Ro, Bundang-Gu, Seongnam, Gyeonggi 13488 Republic of Korea; 3 Division of Hematology, Department of Internal Medicine, Catholic Blood and Marrow Transplantation Center, Seoul St. Mary’s Hospital, The Catholic University of Korea, 222 Banpo-Daero, Seocho-Gu, Seoul, 06591 Republic of Korea

**Keywords:** NK cell line, Immunostimulatory potential, Off-the-shelf platform, Adoptive cancer immunotherapy

## Abstract

**Background:**

Human natural killer (NK) cell lines serve as an attractive source for adoptive immunotherapy, but NK-92 remains the only cell line being assessed in the clinic. Here, we established a novel NK cell line, NK101, from a patient with extra-nodal natural killer/T-cell lymphoma and examined its phenotypic, genomic and functional characteristics.

**Methods:**

Single cell suspensions from lymphoma tissue were expanded with anti-NKp46/anti-CD2-coated beads in the presence of IL-2. A continuously growing CD56^+^ cell clone was selected and designated as NK101. Flow cytometry and RNA sequencing were used to characterize phenotypic and genomic features of NK101. In vitro cytotoxicity and IFN-γ/TNF-α secretion were measured by flow cytometry-based cytotoxicity assay and enzyme-linked immunosorbent assay, respectively, after direct co-culture with tumor cells. Immunomodulatory potential of NK101 was assessed in an indirect co-culture system using conditioned medium. Finally, in vivo antitumor efficacy was evaluated in an immunocompetent, syngeneic 4T1 mammary tumor model.

**Results:**

NK101 displayed features of CD56^dim^CD62L^+^ intermediate stage NK subset with the potential to simultaneously act as a cytokine producer and a cytotoxic effector. Comparative analysis of NK101 and NK-92 revealed that NK101 expressed lower levels of perforin and granzyme B that correlated with weaker cytotoxicity, but produced higher levels of pro-inflammatory cytokines including IFN-γ and TNF-α. Contrarily, NK-92 produced greater amounts of anti-inflammatory cytokines, IL-1 receptor antagonist and IL-10. Genome-wide analysis revealed that genes associated with positive regulation of leukocyte proliferation were overexpressed in NK101, while those with opposite function were highly enriched in NK-92. The consequence of such expressional and functional discrepancies was well-represented in (i) indirect co-culture system where conditioned medium derived from NK101 induced greater proliferation of human peripheral blood mononuclear cells and (ii) immunocompetent 4T1 tumor model where peritumoral injections of NK101 displayed stronger anti-tumor activities by inducing higher tumor-specific immune responses. In a manufacturing context, NK101 not only required shorter recovery time after thawing, but also exhibited faster growth profile than NK-92, yielding more than 200-fold higher cell numbers after 20-day culture.

**Conclusion:**

NK101 is a unique NK cell line bearing strong immunostimulatory potential and substantial scalability, providing an attractive source for adoptive cancer immunotherapy.

**Electronic supplementary material:**

The online version of this article (10.1186/s40425-019-0612-2) contains supplementary material, which is available to authorized users.

## Background

Adoptive cell transfer (ACT) immunotherapy has gained increasing attention in recent years, particularly for the clinical successes of chimeric antigen receptor (CAR)-modified T cell therapies as shown by high complete remission (CR) rates of 70–94% in B cell hematological malignancies [1]. Despite these remarkable results, T cell-based therapies still face numerous challenges for their real-world applications: (i) limited efficacy in solid tumors; (ii) risk of developing cytokine release syndrome (CRS) and long-term side effects; (iii) complex manufacturing and logistics of personalized therapeutics in the autologous settings [2]. To address such unmet needs, natural killer (NK) cells are currently being explored as an alternative platform for ACT based on their unique advantages [3]. Unlike T cells, NK cells do not require prior sensitization for activation and elicit rapid killing activity in a major histocompatibility complex-unrestricted manner, thus displaying broader tumor specificity [4]. NK cells are also considered as safer effector cells since their inability to induce interleukin-6 production [5] and short in vivo lifespan [6] reduce the risks for CRS and persisting toxicities, respectively. Moreover, NK cells can be applied in the allogeneic settings without promoting graft-versus-host disease [7], offering an off-the-shelf treatment option for patients with less complicated and more cost-effective procedures [8]. Accordingly, the utilization of NK cells for ACT would bring significant benefits in terms of efficacy, safety, and patient accessibility.

NK cells used in the clinic are classified into three categories based on their source: (i) patient-derived autologous cells; (ii) healthy donor-derived allogeneic cells; (iii) continuously growing, clonal NK cell lines derived from NK lymphoma/leukemia [6–8]. Allogeneic NK cells are more often used than autologous ones, as NK cells from patients frequently exhibit dysfunctional features, including impaired proliferation, cytotoxicity or cytokine production as well as defective expression of activating receptors or intracellular signaling molecules [9, 10]. Although adoptive transfer of NK cells from killer cell immunoglobulin-like receptor (KIR) ligand-mismatched donors has shown early signs of efficacy in certain settings [7], evidence for clear clinical benefit is still pending. Additionally, primary NK cell-based therapies, either autologous or allogeneic, face operational and technical challenges for large-scale production [2, 6, 7]. Since NK cells represent only 10% of circulating lymphocytes, repeated leukaphereses are required to obtain sufficient cell numbers, causing major inconvenience for patients or donors [7, 8]. Ex vivo expansion of NK cells requires sophisticated protocols for using genetically engineered feeder cells for co-culture and retrieving maximal number of cells without functional impairment [6]. Inter-donor variations and inter-cellular heterogeneity also poses difficulties in standardizing NK cell products [7]. To overcome these limitations, investigators have been trying to employ stable NK cell lines for clinical application [2, 4, 8]. Without needing invasive procedures, clonal NK cell lines consisting of homogenous population can be easily expanded under feeder-free conditions and repeatedly cryopreserved-thawed with minimal loss of viability or functionality [6, 8, 11]. These properties enable clinical scale manufacturing of cellular products with standardized quality [11–13], making NK cells lines an ideal platform for industrialization.

Here, we present a novel NK cell line from a patient with extra-nodal NK/T cell lymphoma and assessed its phenotypic, genomic and functional characteristics. The focus of the study was to identify unique features that distinguish our NK cell line from existing ones and to assess its potential for therapeutic application as an anti-cancer cellular platform.

## Methods

### Case history

A 58-year-old Korean male patient with a history of malignant lymphoma presented with painful erythematous lesions of variable size primarily affecting the lower extremities. A whole body PET/CT showed multiple hypermetabolic lesions. Immunohistochemical analysis of a biopsy demonstrated large atypical cells positive for CD3, CD56 and granzyme B, yielding a diagnosis of extra-nodal NK/T cell lymphoma, nasal type. The patient was initially treated with three cycles of L-asparaginase, cyclophosphamide, vincristine, doxorubicin and dexamethasone (CHOP-L) and achieved a partial remission. However, the patient developed neurological symptoms after three additional cycles of CHOP-L and a subsequent brain biopsy suggested a recurrence of the disease. Immunohistochemical and in situ hybridization analysis revealed the expression of CD56 protein and *EBER* mRNA in the biopsy (Additional file 1: Figure S1). Whole brain radiotherapy was then performed, but the patient died of infections and other treatment complications.

### Establishment and characterization of NK101 cell line

Lymphoma tissue was obtained with the informed consent of a patient and ethical approval by the Institutional Review of Board of the Catholic University of Korea. Detailed procedures for the establishment of NK101 and its phenotypic and functional characterization are described in *Online* Additional file 2.

### Gene expression profiling by RNA-sequencing

Culture expanded NK101 or NK-92 cells were washed twice with phosphate buffered saline (PBS, Hyclone, Logan, UT, USA), pelleted by centrifugation and immediately frozen in liquid nitrogen. Pellets were sent to Theragen Etex Bio Institute (Seoul, Korea) for RNA extraction and whole-transcriptome sequencing by using HiSeq2500 platform (Illumina, San Diego, CA, USA). Transcriptome data was processed according to the institute’s protocol including filtering, sequence alignment through the human reference genome (Ensembl release 72) using the aligner STAR v.2.3.0e, gene expression estimation using Cufflinks v2.1.1, and DEG (differentially expressed gene) analysis.

### Gene set enrichment analysis

Gene Set Enrichment Analysis (GSEA) was employed for the characterization of the entire DEGs identified by RNA-sequencing and performed by using GSEA software v3.0 (http://www.broadinstitute.org/gsea) with the default settings. The DEGs were ranked based on the fold-change, and statistical significance was determined by nominal *p*-value< 0.05 and false discovery rate (FDR) < 0.25. Gene sets of interest were retrieved from the C5 collection (C5.GO biological process gene sets, v6.2) of the Broad Institute Molecular Signature Database. Core genes were identified based on the enrichment scores calculated by the software.

### Animal study

Four- to six-week-old female Balb/c mice were obtained from Genexine Co. Ltd. (Seongnam, Korea), and maintained under specific pathogen-free conditions. Animal experiments were performed in compliance with the guideline of the Institutional Animal Care and Use Committee. For the establishment of a syngeneic mouse tumor model, 1 × 10^6^ cells of 4T1 breast cancer cells expressing enhanced green fluorescent protein (EGFP) and firefly luciferase (fLuc) were injected into Balb/c mice subcutaneously. After palpable tumors formed, the mice were classified into three groups based on tumor size. Serum-free media, 5 × 10^6^ cells of NK-92 or NK101 were infused peritumorally at day 7, 10, 13, and 16. Tumor growth was monitored by size measurement twice a week. At day 21 post tumor injections (PTI), bioluminescence imaging (BLI) was performed by using Lago (Spectral Imaging Instruments, Tucson, AZ, USA) 10 min after subcutaneous injection of 150 mg/kg of D-luciferin (Goldbio, St Louis, MO, USA). For ethical reasons, mice were sacrificed when the tumor size reached over 1000mm^3^.

### Statistics

All data are displayed as mean value ± SD. Differences between the data were evaluated by Student’s t-test by using Graphpad Prism (San Diego, CA, USA). *P*-value less than 0.05 was considered as statistically significant.

For gene expression profiling, statistically significant DEGs between two different NK cell lines were determined using Cufflinks software v2.2.1 where *p*-value was calculated by Cuffdiff algorithm [14] with an approach based on a beta negative binomial model and the t-test for deriving test statistics [15]. The p-value was further adjusted with the Benjamini-Hochberg correction, generating q-value (FDR-adjusted p-value) as previously described [16]. Genes with the q-value less than 0.05 were considered as significant.

## Results

### Establishment of a novel natural killer cell line, NK101

**Fig. 1:**
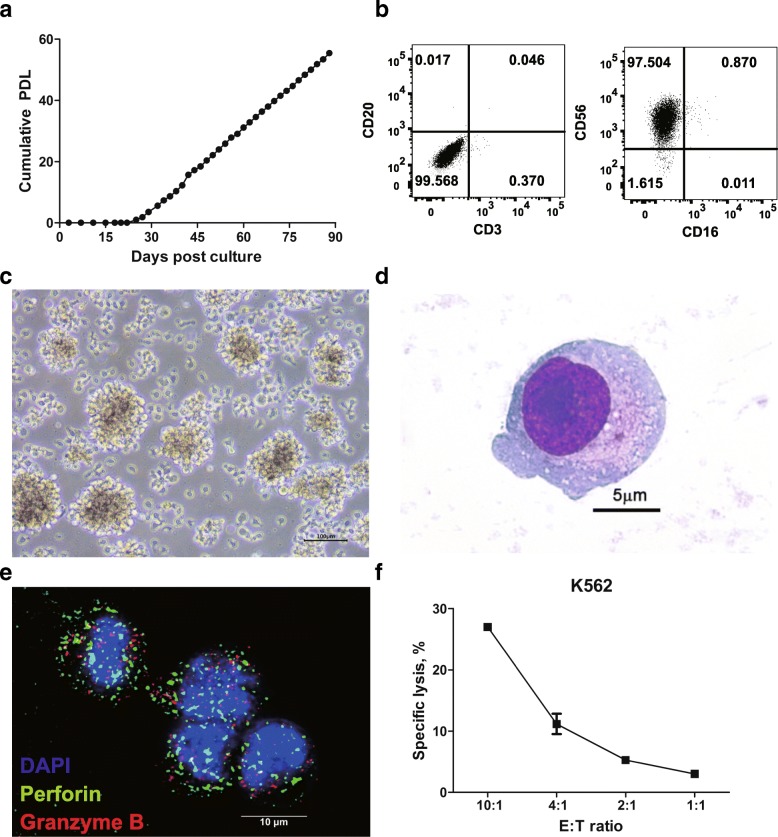
A newly established cell line, NK101, with natural killer cell-like characteristics. **a** Primary mononuclear cells isolated from a patient’s lesion were cultured for more than 90 days. Cell growth is displayed as cumulative population doubling level (PDL) for 90 days. **b** Lineage phenotype of isolated tumor cells was analyzed by flow cytometry. Cells were stained with fluorochrome-conjugated antibodies specific to CD3, CD16, CD20, and CD56. Representative dot plots from 2 independent experiments were displayed after gating singlets and live cells. The numbers indicate the percentage of cells in each quadrant. **c** Growing morphology of NK101 cells in culture is displayed as light microscopic image. 400X magnification. Scale bar = 100 μm. **d** Morphology of a single NK101 cell was visualized under light microscopy after Wright-Giemsa staining. 1000X magnification. Scale bar = 5 μm. **e** Expressions of perforin and granzyme B in NK101 cells were visualized by confocal microscopy after staining with Alexa Fluor 488-conjugated anti-perforin antibody (green), Alexa Flour 647-conjugated anti-granzyme B antibody (red), and DAPI counter-staining (blue). 1000X magnification. Scale bar = 10 μm. **f** NK101 cells were co-cultured with carboxyfluorescein diacetate succinimidyl ester (CFSE)-labeled K562 cells at the indicated effector-to-target (E:T) ratio for 24 h. Apoptotic and dead cell population were discriminated by Annexin-V and fixable viability dye staining, followed by flow cytometric analysis. Percentage of specific lysis percentage was calculated by the formula described in *Online* Additional file 2. Data represent mean ± SD of 3 independent experiments

Primary extra-nodal NK/T cell lymphoma tissue was dissociated into single cells and expanded in the presence of IL-2. After 3 weeks cells began to proliferate and thereafter maintained stable growth kinetics over a period of 3 months (Fig. 1a). The majority of cells were negative for CD3 and CD20, but positive for CD56 (Fig. 1b). CD56^+^ population was sorted as single cells and a clone with stable growth profile was selected and designated as NK101. NK101 cells were free of bacterial or viral infections (Additional file 3: Table S1), except for Epstein-Barr virus (EBV). Although *EBNA-2* latency gene was detected by PCR with genomic DNA of NK101 (Additional file 1: Figure S2a), expression of a lytic protein BZLF1 was not detected by Western blotting even after stimulation with sodium butyrate and PMA (Additional file 1 Figure S2b). These data suggest that NK101 is latently infected with EBV but do not produce active virions, yielding similar results with NK-92 [17]. NK101 cells grew as multicellular aggregates, as with previous studies on NK-92 and NKG [18, 19] (Fig. 1c). NK101 cells appeared to present LGL morphology (Fig. 1d) and expressed perforin and granzyme B as shown by immunofluorescence microscopy (Fig. 1e). NK101 was also capable of killing K562 cells in an effector-to-target ratio-dependent manner, indicating MHC-unrestricted cytotoxicity (Fig. 1f). Collectively, these results suggest that NK101 possessed fundamental characteristics of NK cells.

### Immunophenotypic analysis of NK101

**Fig. 2:**
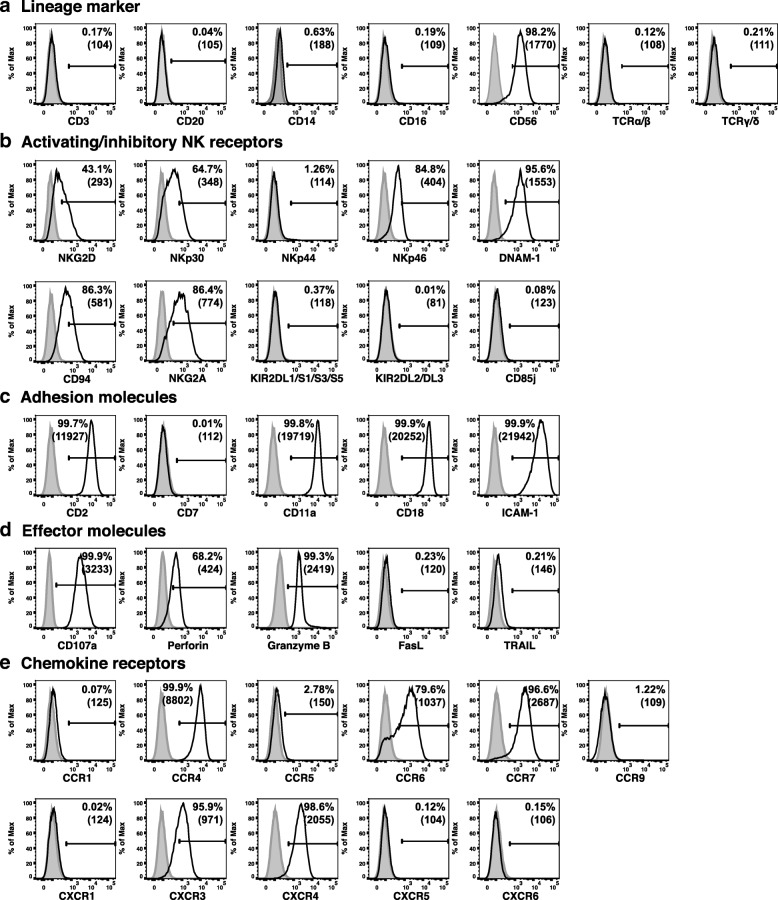
Immunophenotype analysis of NK101. Expression of indicated surface or intracellular markers on cultured NK101 cells was analyzed by flow cytometry**.** Markers were classified into 5 categories including **a** lineage markers, **b** activating/inhibitory NK receptors, **c** adhesion molecules, **d** effector molecules, **e** chemokine receptors. Gray-filled histogram indicates isotype control, while open histogram indicates each marker expression. Results are representative of 3 independent experiments. Numbers in the histograms and parentheses indicate the percentages and mean fluorescence intensity of the gated population, respectively

Flow cytometric immunophenotypic analysis was performed to understand the lineage and differentiation/activation status of NK101. Expression analysis of lineage markers revealed positive staining for CD56 but not CD3, CD20, CD14, CD16, TCRαβ or TCRγδ, suggesting NK cell origin of NK101 [20] (Fig. 2a). It was also noted that CD16 was absent on NK101, similar to other NK cell lines [8, 20]. Among killer activation receptors, NKG2D, NKp30, NKp46, and DNAM-1, but not NKp44, were expressed on NK101 cells. In terms of killer-cell immunoglobulin-like receptors, NK101 was negative for KIR2DL1/DL2/DL3, KIR2DS1/DS3/DS5 and CD85j (ILT-2), but positive for CD94 and NKG2A (Fig. 2b). Although such expression of a broad range of activating receptors and relative lack of inhibitory receptors resembled those of NK-92 [21], distinguishing features of NK101 were the presence of DNAM-1 and the absence of ILT-2. Adhesion molecule analysis showed that NK101 cells expressed high levels of CD2, CD11a, CD18, and ICAM-1, but a negligible level of CD7 (Fig. 2c). Positive expression of CD107a, perforin and granzyme B served as an indicator of cytotoxic potential of NK101, whereas negative expression of Fas ligand (FasL) and tumor necrosis factor-related apoptosis-inducing ligand (TRAIL) indicating the lack of potential to mediate death receptor-mediated apoptosis (Fig. 2d). For chemokine receptors, NK101 cells showed positive expression of CCR4, CCR6, CCR7, CXCR3 and CXCR4 with negative expression of CCR1, CCR5, CCR9, CXCR1, CXCR5 and CXCR6 (Fig. 2e).

### Prediction of developmental origins of NK101

**Fig. 3:**
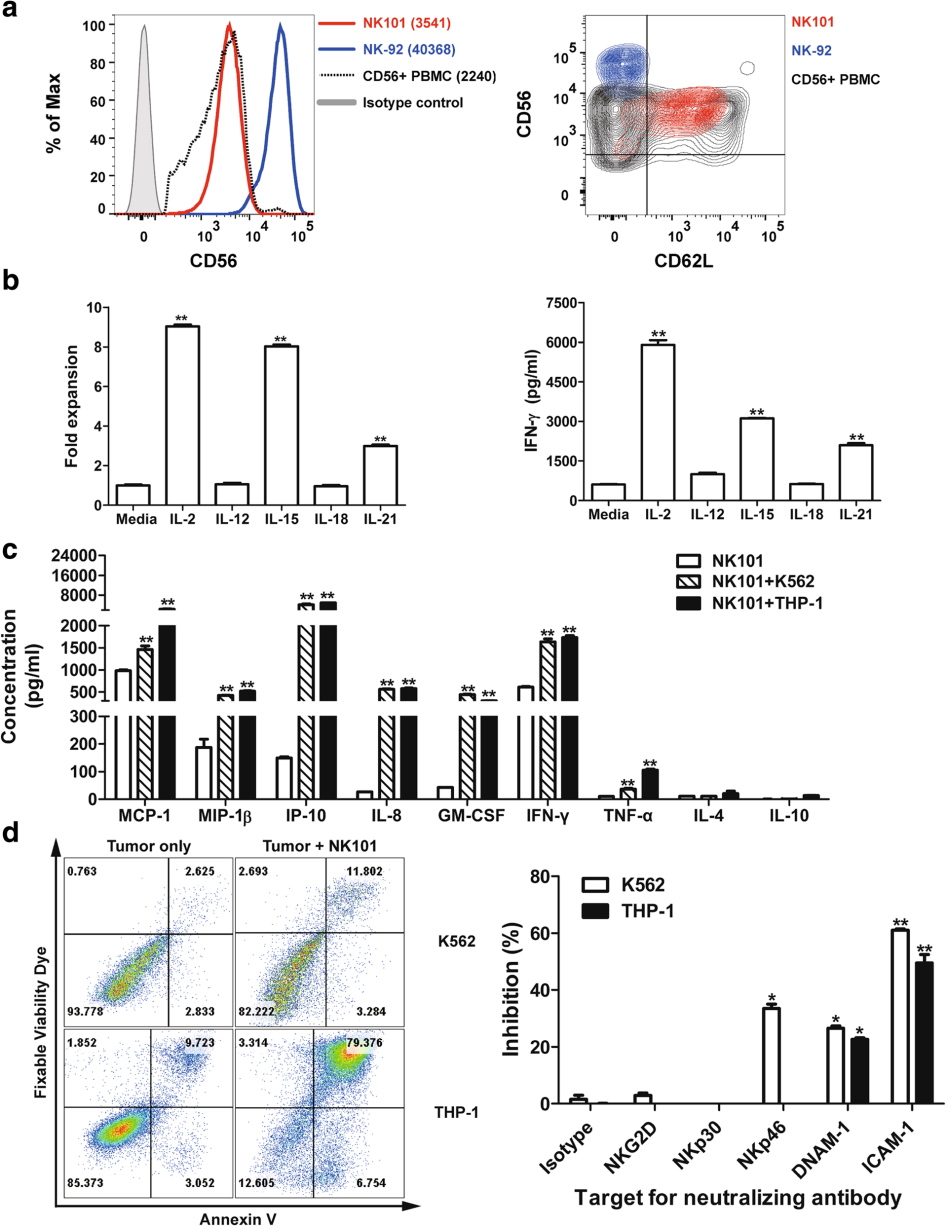
CD56^dim^CD62L^+^ NK-like features of NK101. **a** NK101, NK-92 and primary human peripheral blood mononuclear cells (PBMCs) were stained with fluorochrome-conjugated anti-CD3, −CD19, −CD56, and -CD62L antibodies. Results are displayed as one-color histogram (left) or two-color contour (right) plots after gating live, CD3^−^, CD19^−^, and CD56^+^ cells. Red, blue and black indicate NK101, NK-92, primary CD56^+^ NK cells, respectively. In the histogram plot, gray-filled line represents isotype control and the numbers in the parenthesis represent geometric mean fluorescence intensity of CD56 (left). Results are representative of 3 independent experiments. **b** Cultured NK101 cells were treated with 10 ng/ml of indicated cytokines (except IL-2; 500 IU/ml). Cell expansion was assessed by MTS assay after 3 days (left) and IFN-γ secretion was measured by ELISA after 24 h (right). Data represent mean ± SD of triplicate wells from 3 independent experiments. **p < 0.01 versus the corresponding untreated groups. **c** The secretion of indicated cytokines or chemokines from rested, K562-(E:T = 4:1), or THP-1-(E:T = 4:1) co-cultured NK101 cells was measured by a multiplex immunoassay. Data represent mean ± SD of triplicate wells from 2 independent experiments. **p < 0.01 versus rested NK101. **d** NK101 cells were co-cultured with CFSE-labeled K562 or THP-1 cells at an effector-to-target ratio of 4:1 for 24 h in the absence (left) or presence of indicated neutralizing antibody (10 μg/ml) (right). Harvested cells were stained with Annexin-V and fixable viability dye, and CFSE^+^ tumor cells were analyzed by flow cytometry. Representative plots from 3 independent experiments are shown (left). Bar graphs represent mean ± SD of triplicate wells from 3 independent experiments (right). *p < 0.05, **p < 0.01 versus corresponding isotype control groups

The conventional model of NK cell differentiation has described that CD56^bright^ cells are immature precursors whereas CD56^dim^ cells are terminally differentiated progeny [22]. Comparative analysis of CD56 expression level revealed that NK-92 was CD56^bright^, in agreement with previous findings [18, 21], while NK101 was CD56^dim^ (Fig. 3a, left), displaying differentiated phenotype. Since CD62L is known to be a marker for intermediate stage [23], we investigated CD62L expression level in NK101. As a result, high level expression of CD62L was found in NK101, but not in NK-92 (Fig. 3a, right), implicating that NK101 cells were arrested at the middle stage of differentiation process from CD56^bright^ to CD56^dim^ NK cells. Next, we investigated whether NK101 retained functional properties of a CD56^dim^CD62L^+^ NK cells in terms of (i) proliferation and IFN-γ secretion upon cytokine stimulation (ii) cytokine production and target killing upon activating receptor engagement as previously reported [23]. Firstly, NK101 cells were treated with different cytokines and the extent of cell expansion as well as IFN-γ induction was measured. The cell number retrieved 3 days after IL-2, IL-15 and IL-21 treatment was 9-fold, 8-fold and 3-fold higher than untreated control, respectively. IL-12 and IL-18 failed to promote the proliferation (Fig. 3b, left). Similarly, IL-2, IL-15, and IL-21 treatment significantly increased IFN-γ secretion from NK101, but not with IL-12 and IL-18 (Fig. 3b, right). Secondly, we assessed the secretion of various chemokines and cytokines from NK101 in response to co-culture with K562 or THP-1 cells. In comparison to unstimulated cells, NK101 cells in both K562 and THP-1 co-cultures showed increased expression of MCP-1, MIP-1β, IP-10, IL-8, GM-CSF, IFN-γ and TNF-α (Fig. 3c). Lastly, we investigated whether tumor cell killing by NK101 is mediated by activating receptor engagement. Again, K562 and THP-1 were selected as target tumor cell lines, displaying low (14%) and high (84%) susceptibility to killing by NK101 cells, respectively (Fig. 3d, left). We then treated blocking antibodies against representative activating receptors (NKG2D, NKp30, NKp46 or DNAM-1) and an adhesion molecule (ICAM-1) in NK101/tumor cell co-cultures. As a result, we found significant inhibition of NK101 cytotoxicity by anti-DNAM-1 and anti-ICAM-1 antibodies in both co-cultures. Anti-NKp46 antibody exerted inhibitory effect in K562 co-culture alone (Fig. 3d, right). These results not only provide a direct evidence for activating receptor engagement-mediated NK101 cytotoxicity, but also suggest simultaneous involvement of multiple receptors in triggering cytolytic pathway of NK cells as previously described [24, 25]. Overall, NK101 seemed to retain both phenotypic and functional characteristics of CD56^dim^CD62L^+^ NK subset.

### Comparison of effector functions between NK101 and NK-92

NK-92 has been known to possess the strongest cytotoxicity among established human NK cell lines [2]. To assess anticancer potential of NK101 relative to NK-92, head-to-head comparison of the cytotoxicity and effector molecule secretion was performed.

**Fig. 4:**
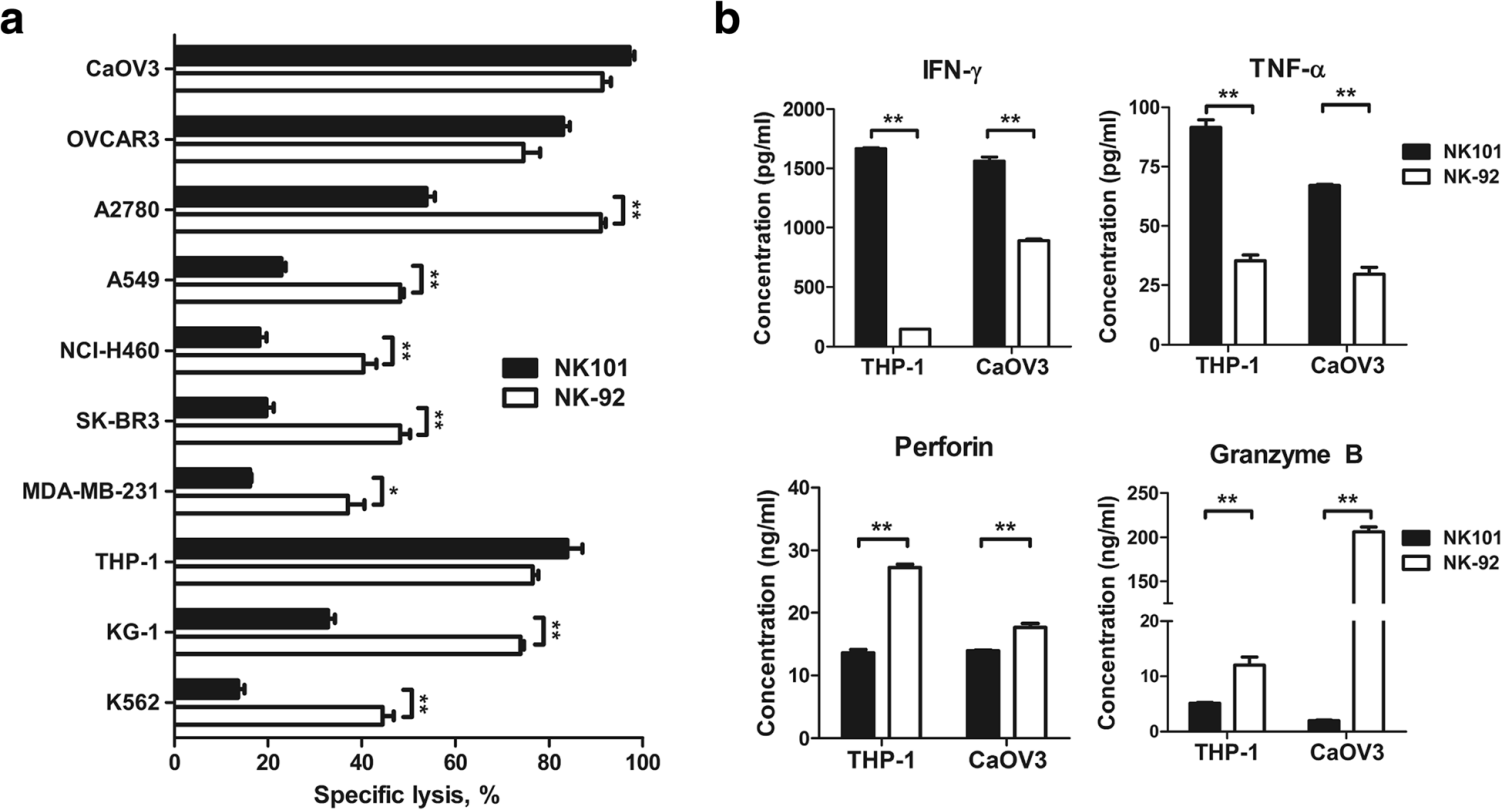
Comparative analysis of the cytotoxicity and effector molecule secretion by NK-92 and NK101. **a** Indicated cell lines were co-cultured with NK101 (black bar) and NK-92 (white bar) at an effector-to-target ratio of 4:1 for 24 h. The percentages of apoptotic tumor cells were quantified by Annexin-V and fixable viability dye staining via flow cytometry. Percentage of specific lysis was calculated by the formula described in *Online* Additional file 2. *p < 0.05, **p < 0.01. **b** IFN-γ, TNF-α, perforin, and granzyme B concentrations in the co-culture supernatants were determined by ELISA. All data represent mean ± SD of 3 independent experiments. **p < 0.01

Firstly, we co-cultured NK101 or NK-92 with human tumor cell lines of various tissue origin and measured apoptosis of tumor cells after 24 h. NK101 exerted similar level of cytotoxicity against 2 out of 3 ovarian cancer cell lines tested - CaOV3 and OVCAR3. On the other hand, NK-92 was more potent in killing lung cancer (A549, NCI-H460) and breast cancer (MDA-MB-231, SK-BR3) cell lines. Against blood cancer cell lines, NK101 displayed comparable cytotoxicity against THP-1 but less effective at killing KG-1 and K562 compared to NK-92 (Fig. 4a). Overall, NK101 seemed to possess weaker in vitro cytotoxicity than NK-92, which exhibited more consistent and reproducible killing as reported [2]. A similar trend was shown in co-cultures with murine tumor cell lines (Additional file 1: Figure S3).

Secondly, we analyzed the amount of key effector molecules – IFN-γ, TNF-α, perforin and granzyme B – that are induced by NK101 or NK-92 after tumor cell co-culture. Assessment was performed in THP-1 and CaOV3 co-cultures where similar degrees of tumor cell killing were observed. Interestingly, despite comparable cytotoxicity exerted by NK101 and NK-92, their secretion pattern of effector molecules was markedly different. In THP-1 co-culture, compared to NK-92, NK101 induced 11.5-fold higher IFN-γ, 2.6-fold higher TNF-α, 2.0-fold lower perforin and 2.4-fold lower granzyme B. Analogous pattern of effector molecule secretion was found in CaOV3 co-culture, as shown with 1.8-fold higher IFN-γ, 2.3-fold higher TNF-α, 1.3-fold lower perforin and 103.5-fold lower granzyme B induction by NK101 (Fig. 4b). These results implicate that NK101 engages different mechanism of tumor cell killing from NK-92 and IFN-γ/TNF-α might play a greater role than perforin/granzyme B in NK101 cytotoxicity.

### Comparison of immunomodulatory potential based on differential gene expression and cytokine secretion profiles between NK101 and NK-92

**Fig. 5:**
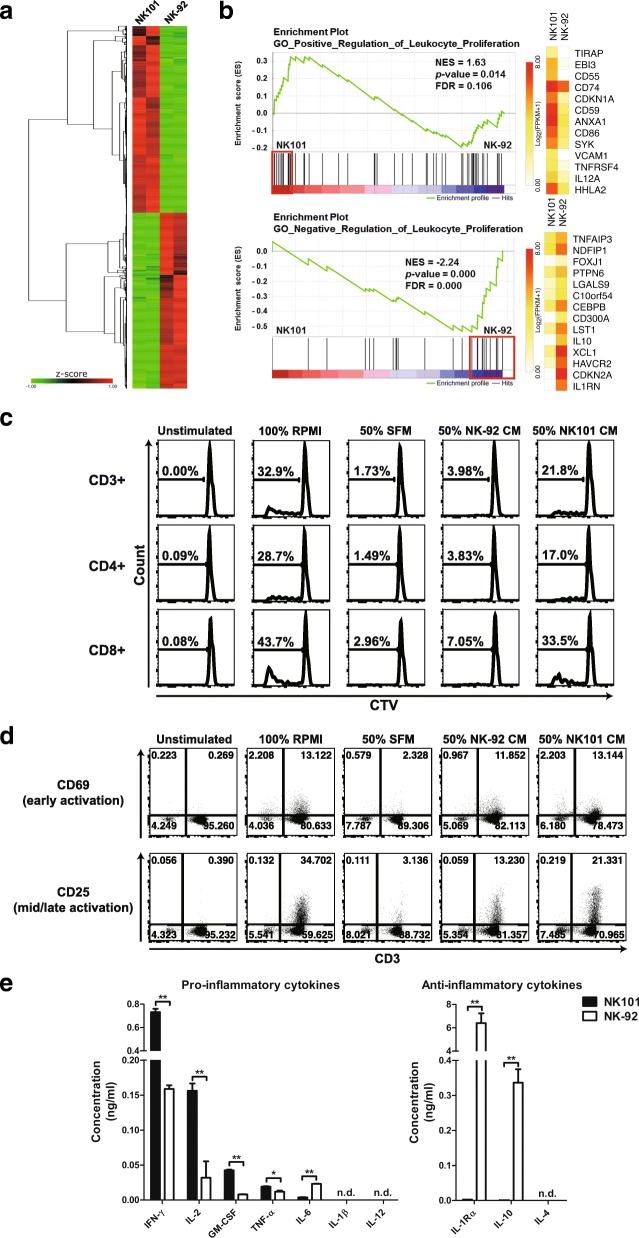
Comparative analysis of molecular expression profiles and immunostimulatory potential between NK101 and NK-92. **a** A heat map displaying upregulated (red) and downregulated (green) genes is shown (q < 0.05). Genes were clustered by one minus Pearson correlation with complete linkage algorithm. Signal levels are shown as z-score color key. **b** Gene set enrichment analysis (GSEA) was performed in terms of ‘positive regulation of leukocyte proliferation’ (top) or ‘negative regulation of leukocyte proliferation’ (bottom), then the enrichment plots were illustrated with normalized enrichment score (NES), *p*-value, and false discovery rate (FDR). Core genes for each term were marked by red boxes, and their expression levels were exhibited as heatmaps. A white-yellow-red color scale indicates expression level of each gene transformed as Log_2_ (FPKM+ 1). **c, d** Human PBMCs labeled with CellTrace Violet (CTV) were either unstimulated or stimulated with anti-CD3 and cultured under indicated conditions for 5 days. Total cells were stained using Live/Dead Fixable Viability Dye and fluorochrome-conjugated antibodies specific to either CD3/CD4/CD8 **(c)** or CD3/CD25/CD69 **(d)**. **c** Representative histograms for CTV in the gate of CD3+, CD4+, and CD8+ cells are shown. **d** Representative dot plots for CD69 and CD25 expression are exhibited in terms of CD3+ population after live cell gating. The results are representative of 2 independent experiments from a single donor in triplicate. SFM, serum-free medium; CM, conditioned medium. **e** The concentrations of indicated pro- and anti-inflammatory cytokines in CM derived from NK101 (black bars) or NK-92 (white bars) were measured by an individual ELISA kit. Data represent mean ± SD of triplicate wells from 2 independent experiments. *p < 0.05, **p < 0.01; n.d., not detected

A recent study of genome-wide analyses of human NK cell lines has provided deeper understanding of their origins and the source of functional discrepancies [26]. Similarly, we performed RNA-sequencing to compare gene expression profile of NK101 and NK-92 (Additional file 4: TableS2). Although both cell lines were derived from NK cell lymphoma, NK101 and NK-92 demonstrated quite distinct gene expression profiles (Fig. 5a). Over 20,000 genes evaluated, 5187 DEGs were identified, with 2696 up- and 2491 down-regulated genes in NK101 compared to NK-92. Nextly, we employed GSEA to evaluate genetic signatures associated with DEGs. NK101 showed a significant enrichment of core genes involved in ‘positive regulation of leukocyte proliferation’, while NK-92 displayed a strong enrichment of core genes involved in ‘negative regulation of leukocyte proliferation’ (Fig. 5b). These results suggest that NK101 expresses more genes with potential immunostimulatory properties. It was also worth noting that NK-92 exhibited a relative enrichment of genes associated with ‘cytolysis’ including *PRF1 and GZMA* (Additional file 1: Figure S4).

We next tested whether NK101 indeed possesses superior capacity to induce leukocyte proliferation using an indirect co-culture system [27]. Methodically, human peripheral blood mononuclear cells (PBMCs) were stimulated with anti-CD3 in the presence of NK101- or NK-92-conditioned medium (CM) or control serum-free medium (SFM), cultured for five days, and their proliferation was measured by the degree of CellTrace Violet (CTV) dilution. While about 30% of whole PBMC population proliferated in 100% RPMI condition, the addition of SFM failed to support their proliferation. Notably, NK-92-CM minimally supported the growth of responder cells, whereas NK101-CM markedly enhanced their proliferation, as shown by 5-fold higher whole PBMCs, 4-fold higher CD3- population and 5-fold higher CD3+ T cell population that were CTV-low compared to corresponding NK-92 CM treated groups. As with 100% RPMI-treated groups, CD8+ T cells tend to show superior proliferation than CD4+ T cells in both NK101-CM and NK-92-CM treated groups (Fig. 5c and Additional file 1: Figure S5). Furthermore, we assessed the expression levels of early (CD69) and mid-to-late (CD25) T cell activation markers [28] on CD3+ lymphocytes. In line with the results for proliferation, NK101-CM treated T cells showed higher CD25 expression than NK-92 CM treated counterparts. We did not find much difference in CD69 expression between two groups on day 5 (Fig. 5d). Overall, these results provide direct evidence for superior immunostimulatory potential of NK101 over NK-92.

Immunomodulatory effects of cellular therapeutics are controlled by the balance between their production of pro-inflammatory and anti-inflammatory cytokines [29]. We therefore measured the concentration of those cytokines in CM-derived from NK-92 or NK101 via ELISA. In terms of pro-inflammatory cytokine expression, NK101-CM contained 4.6-fold higher IFN-γ, 5.3-fold higher GM-CSF, 4.9-fold higher IL-2 and 1.6-fold higher TNF-α than did NK-92-CM. IL-6 was the only cytokine that was produced in lower amounts compared to NK-92 (Fig. 5e, left). On the other hand, the amount of anti-inflammatory cytokines, IL-1 receptor antagonist (IL-1ra) and IL-10, in NK101-CM was 2423-fold and 692-fold lower than NK-92-CM, respectively (Fig. 5e, right). To validate the expression profiles for above-mentioned cytokines at the gene levels, relative RNA levels in NK101 and NK-92 cells were quantified via real time-PCR. In correlation with ELISA results, RNA expression levels of GM-CSF was higher while those of IL-6, IL-1ra and IL-10 were lower in NK101 cells compared to NK-92 cells. Contrarily, RNA expression level of IFN-γ was similar and IL-2 and TNF-α was even lower in NK101 cells, showing opposite pattern of expression from ELISA (Additional file 1: Figure S6). Although the lack of correlation between RNA and protein concentration in cells has been occasionally reported [30], we asked whether expression patterns of certain cytokines could be upregulated in secretome (supernatant) and downregulated in intracellular proteome (cell lysates) as previously described [31]. Interestingly, we found that expression of IFN-γ and IL-32α were much higher in NK101 supernatant, while significantly lower in NK101 cell lysates than corresponding NK-92 counterparts (Additional file 1: Figure S7). Therefore, the lack of correlation in gene and protein expression levels of certain cytokines could also be originated from the source of detection (secretome vs. intracellular proteome).

### Comparison of in vivo antitumor efficacy of NK101 and NK-92 in immunocompetent mice

Immunodeficient xenograft models would not accurately predict the efficacy of NK101 in human as the aspects of cytokine-induced modulation of tumor microenvironment and activation of host immunity are not reflected. Since NK101 was characterized by its distinct ability to produce immunostimulatory cytokines, we aimed to evaluate antitumor efficacy of NK101 in immunocompetent mice bearing syngeneic tumors. 4T1 mammary carcinoma was selected for allograft as it was most susceptible to NK101-mediated cytolysis among murine cell lines tested (Additional file 1: Figure S3).

**Fig. 6:**
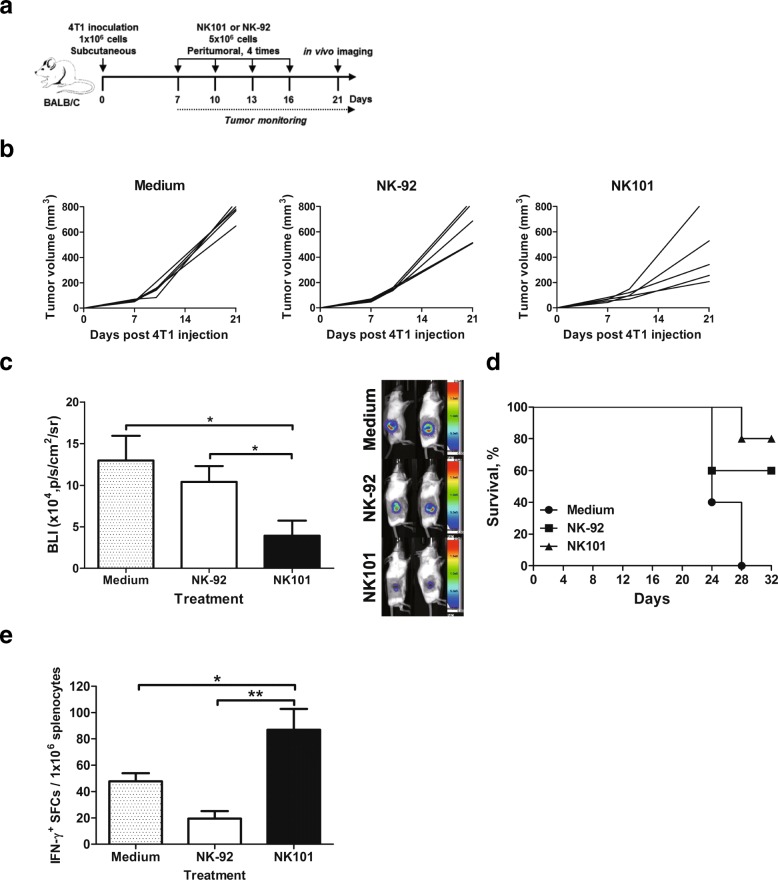
Anti-tumor effects of NK101 and NK-92 in immunocompetent 4T1 tumor model. **a** Experiment schema: Balb/c mice were injected with 1 × 10^6^ cells of 4T1 expressing EGFP-fLuc cells subcutaneously. After palpable tumors had formed, the mice were grouped based on tumor size. 5 × 10^6^ cells of NK-92 or NK101 were injected peritumorally for 4 times at days 7, 10, 13 and 16. Tumor size was monitored for 3 weeks (**b**) and bioluminescent imaging was performed on day 21 (**c**). **b** The change of tumor size in the individual mice over time was represented by a line. **c** Bioluminescent signals were quantitated using Amiview and plotted as bar graph. Data represent mean ± SD of 5 mice per group from 2 independent experiments (left). Representative tumor images in each group are also shown (right). **d** Kaplan-Meier survival curve of 4T1-bearing mice treated by serum-free media, NK-92 or NK101 is shown (*n* = 5, representative of 2 independent experiments). **e** Splenocytes from tumor-bearing mice treated with serum-free media, NK-92, or NK101 were prepared for IFN-γ ELISPOT assay. Cells were stimulated by 50 μg/ml of tumor lysates for 24 h. The frequency of IFN-γ^+^ spot forming cells (SFCs) per 10^6^ splenocytes is displayed. Data represent mean ± SD of triplicate wells from 2 independent experiments. *p < 0.05, **p < 0.01

Balb/c mice were subcutaneously injected with luciferase expressing 4T1 cells, grown until palpable tumors formed and then infused with NK101 or NK-92 cells peritumorally four times at 3-day intervals (Fig. 6a). Surprisingly, NK101 therapy delayed tumor growth more effectively than NK-92 or medium control (Fig. 6b). On day 21 post tumor injection, bioluminescence signal intensity was about two-fold lower in the NK101 treated group compared to NK-92 (Fig. 6c). On day 32, all medium-treated control mice died while 80 and 60% of mice survived in the groups treated with NK101 or NK-92, respectively (Fig. 6d). We then conducted ELISPOT using splenocytes to measure antigen-specific T cell responses induced by NK101 or NK-92 therapy. Notably, NK101 treatment induced significantly higher numbers of IFN-γ secreting cells than did NK-92 or medium treatment (Fig. 6e). Therefore, superior in vivo efficacy of NK101 over NK-92 was likely to involve the mobilization of host antitumor immunity since direct cytotoxicity of NK101 was weaker than NK-92 (Additional file 1: Figure S3).

### Comparison of proliferative potential of NK101 and NK-92

**Fig. 7:**
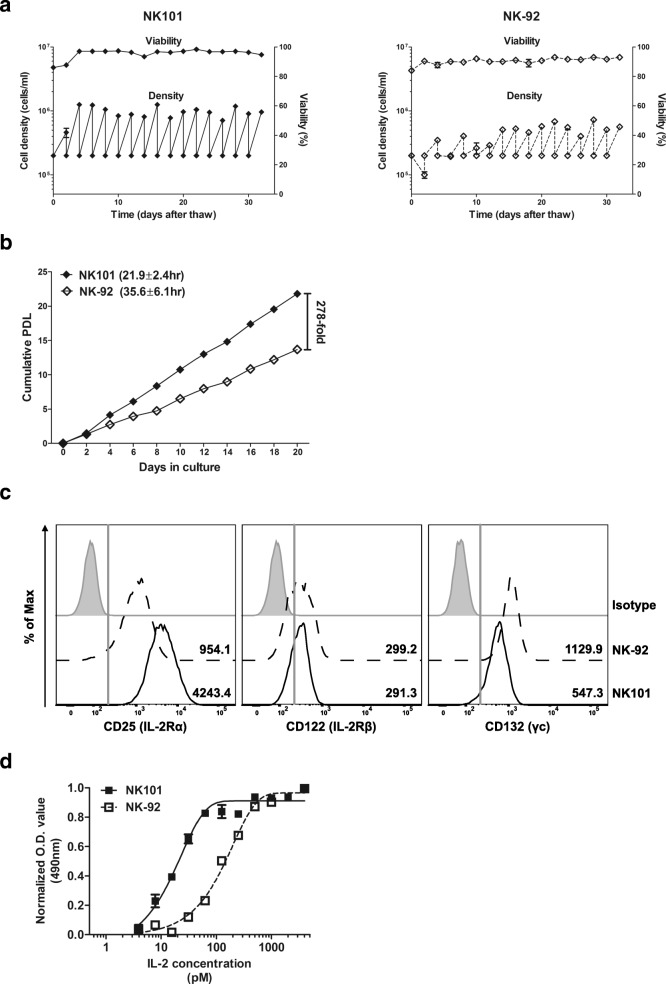
Comparative analysis of expansion capacity of NK101 and NK-92. **a** NK101 and NK-92 cells were thawed from frozen vials and cultured in SCGM media supplemented with 20% FBS and 500 IU/ml of recombinant IL-2 for 32 days. Cells were subcultured every 2 days. Seeding and harvest cell density (lower dots and line, cells/ml) and viability (upper dots and line, %) are displayed together. Data represent mean ± SD of 3 independent experiments. **b** NK101 and NK-92 cells under stable growing conditions were seeded at a density of 2x10^5^cells/ml and cultured for 20 days. Cells were harvested every two days and counted. Cumulative PDL was calculated by the formula described in *Online* Additional file 2. The numbers in the parenthesis indicates doubling time (Td). Data represent mean ± SD of duplicate wells from 2 independent experiments. **c** NK101 and NK-92 cells were stained with PE-conjugated anti-CD25, −CD122, and -CD132 antibodies, and analyzed by flow cytometry. Representative histogram plots from 3 independent experiments were displayed after gating singlets and live cells. Gray-shaded, dotted, and bold line indicates isotype control, NK-92, and NK101, respectively. The numbers in the histogram indicate mean fluorescent intensity. **d** NK101 or NK-92 cells were deprived of IL-2 for 24 h, and then treated with various concentration of IL-2 for 3 days. Expansion of cells was assessed by MTS assay, and the absorbance at 490 nm is normalized into 0 to 1 based on the minimum and maximum values for each cell line. Each dot represents mean ± SD of triplicate wells of two independent experiments

Previous NK-92 manufacturing protocols for clinical studies were composed of the following steps: (i) thawing of cryopreserved cell banks; (ii) expansion for 9–21 days; (iii) harvest and washing; (iv) irradiation and infusion [11, 13, 32, 33]. Consequently, maximum expandable dose was set based on the expansion capacity of thawed NK-92 cells over 2–3 weeks of culture [11]. We therefore compared the growth profile of NK101 and NK-92 after thawing in the same culture conditions. The viabilities of both cell lines were maintained over 80% throughout entire culture period. Two days after thawing, the number of NK-92 cells decreased while that of NK101 cells increased. Moreover, NK101 exhibited stable growth profile from passage 2, whereas NK-92 required additional 5 passages (10 days) to achieve consistent growth rate (Fig. 7a). We then assessed expansion potential of NK-92 and NK101 after they reached stable growth profile. Under our culture conditions utilizing traditional tissue culture flasks, NK-92 cells grew with a doubling time of 35.6 ± 6.1 h displaying a similar rate of expansion with a prior study using Vuelife culture bags [11]. On the other hand, NK101 exhibited more rapid growth with a doubling time of 21.9 ± 2.4 h. Considering typical manufacturing period of 15–21 days for NK-92, NK101 showed 278-fold higher expansion than did NK-92 after 20 days in culture (Fig. 7b). Since the proliferation of NK cells is regulated by IL-2 concentration and the expression of its receptors [34, 35], we compared expression levels of CD25 (IL-2Rα), CD122 (IL-2Rβ) and CD132 (γc) on NK-92 and NK101 via flow cytometry. NK101 displayed similar level of CD122 expression, but lower CD132 expression than NK-92. But interestingly, NK101 showed significantly higher expression of CD25 than NK-92, as represented by 4.4-fold higher mean fluorescence intensity value (Fig. 7c). As higher CD25 expression correlates to greater sensitivity to IL-2 induced proliferation [36], we treated different doses of IL-2 and evaluated the responses by NK101 and NK-92. NK101 began to grow at IL-2 dose of about 8pM and showed saturated growth from 500pM (EC_50_ = 23.3pM), while NK-92 started to grow at IL-2 dose of 30pM and displayed saturated profile from 2000pM (EC_50_ = 128.3pM) (Fig. 7d). These results implicate that NK101 can be expanded in larger scale than NK-92 over the same duration of culture, even with lower IL-2 supplementation.

## Discussion

Eight clonal NK-cell lines have been established over the last two decades, but only one cell line, NK-92, has entered clinical trials for safety and efficacy assessment [2, 3]. To date, a total of 39 patients with advanced cancers in three different phase 1 trials were treated with ex vivo-expanded NK-92 cells, with nearly half of them receiving multiple dosing regimens [11, 13, 33]. The treatment was safe and well-tolerated as shown by the absence of dose-limiting toxicities and one grade 4 adverse event associated with tumor lysis syndrome [33]. However, despite previous evidence proposing NK-92 as the strongest effector among existing NK cell lines [2], NK-92 infusions yielded an objective response rate of only 5% (2 CRs out of 39 patients treated [11, 13, 33]), leaving much room for improvement in terms of therapeutic efficacy. In addition, the highest dose level set based on the expansion capacity of NK-92 cells over 2–3 weeks of manufacturing might have been insufficient to induce meaningful antitumor effects [11]. Accordingly, there remain substantial need for developing an alternative NK cell line with improved efficacy and superior scalability for clinical utilization. In this study, we established a novel human NK cell line, NK101, from a patient with NK/T cell lymphoma and identified phenotypic, genomic and functional features distinguishing NK101 from NK-92, and finally, proposed potential advantages of utilizing NK101 for clinical application as an alternative platform for cancer immunotherapy.

A traditional model of NK cell differentiation utilizes CD56 brightness to define the maturation status and function of NK cells: (i) CD56^bright^ immature, immunoregulatory NK cells with high cytokine producing potential; (ii) CD56^dim^ mature NK cells with strong cytotoxicity; (iii) CD56^dim^CD62L^+^ middle stage NK cells bearing both effector functions [23]. According to this model, however, CD56^bright^-like NK-92 would display lower cytotoxicity and higher IFN-γ production than NK101 with CD56^dim^CD62L^+^ phenotype. Ironically, our study presented opposite results. A recent study comparing genetic, phenotypic and functional characteristics of multiple NK cell lines also showed that CD56^bright^ NK-92 exerted much higher tumor cell killing than CD56^dim^ NKL, and identified *NCAM1* (encoding CD56) as the most differentially expressed gene between lytic and non-lytic cell lines [26]. All of these findings, those of others and ours as well, led us to challenge the conventional notion of CD56 expression and its correlation to NK cell functionality.

A growing body of evidence suggests that CD56^bright^ NK cells exist in two interconverting functional states – anti-inflammatory/regulatory CD56^bright^ and pro-inflammatory/cytotoxic CD56^bright^ cells – and CD56 brightness marks an elevated potential for activation and function in both contexts [37]. It is suggested that ex vivo expansion or cytokine priming can convert weakly cytolytic decidual or tumor-associated CD56^bright^ cells into those with high cytotoxic potential, surpassing that of CD56^dim^ subset [37–39]. Our analysis of gene expression profiles and cytokine production implicated that NK-92 cells are intrinsically decidual-like or tumor-associated CD56^bright^-like, considering its significant enrichment of genes associated with ‘negative regulation of leukocyte proliferation’ and high level secretion of IL-10 and IL-1ra, representative anti-inflammatory and immunosuppressive cytokines [40]. Decidual NK-like characteristics and IL-10 production by NK-92 were also previously described [41]. However, continuous exposure to IL-2 (crucial for NK-92 growth) and ex vivo expansion seemed to shift and maintain NK-92 into highly-activated, cytotoxic CD56^bright^ state with strong degranulation potential. It was also important to note that, unlike IL-15 primed or ex vivo expanded primary CD56^bright^ cells [38, 39], anti-inflammatory or regulatory properties remained unchanged in NK-92. Contrarily, NK101 was genetically programmed to possess immunostimulatory characteristics with a high propensity for pro-inflammatory cytokine production. Although the translation of primary NK cell biology to NK cell lines must be taken cautiously, CD56^bright^ NK-92 seemed to be located the far ends of NK cell functional spectrum possessing anti-inflammatory properties and high cytotoxicity at the same time, while CD56^dim^CD62L^+^ NK101 seemed to occupy intermediate position of pro-inflammatory and cytotoxic section [37].

Numerous studies of novel NK cell lines have compared in vitro cytotoxicity of respective cell lines with NK-92, but none of them evaluated their relative in vivo efficacies. Given the lower in vitro cytotoxicity of NK101, it seemed evident that NK101 would exert weaker antitumor effects than NK-92 in immunodeficient models. However, distinguishable pattern of pro- and anti-inflammatory cytokine secretion as well as differential capacity to induce proliferation and activation of human T cells raised the need to reflect the effects of bystander immune cell activation and their contribution to the overall antitumor efficacy. We therefore proceeded to compare in vivo therapeutic efficacy of NK101 and NK-92 in syngeneic mouse model of 4 T1 breast cancer. Unexpectedly, repeated local administration of NK101 induced greater tumor growth inhibition with concomitant generation of higher tumor-specific T cell responses than did that of NK-92. Considering weaker in vitro cytotoxicity of NK101 against 4 T1, we presumed that the activation of systemic antitumor immunity as well as the formation of more favorable pro-inflammatory tumor microenvironment by NK101 administration contributed to this phenomenon. It is worth emphasizing that IL-2, TNF-α (pro-inflammatory cytokines upregulated in NK101), IL-1 receptor antagonist and IL-10 (anti-inflammatory cytokines upregulated in NK-92) are indeed biologically active in mice [42–45]. To our knowledge, this is the first experimental study to show superior in vivo efficacy of a novel NK cell line compared to NK-92, and more importantly, to validate the contribution of immunomodulatory factors in anti-tumor immunity and overall efficacy.

Large scale manufacturability is a key success factor in the commercialization of cell therapy products [46]. Although NK-92 has been regarded as an off-the-shelf platform providing unlimited source of effector cells, earlier NK-92 manufacturing protocols have been restricted to a single-patient batch scale [47]. For instance, Arai et al. utilized 1-L Vuelife culture bags to produce 1 × 10^9^ cells/bag from initial 6.25 × 10^6^ cells over 15–17 days of culture, resulting in 218~250 folds expansion. However, patients in the highest dose group required about 6 × 10^9^ cells (3 × 10^9^ cells/m^2^) per dose, requiring six 1-L bags. This also means that a single production cycle yielded only 0.17 dose [33]. In a separate study, Tonn et al. used larger bags to produce 1~2 × 10^10^ cells over 9–12 days, but patients in the highest dose group required about 2 × 10^10^ cells (1 × 10^10^ cells/m^2^) per dose. Therefore, only 0.5–1 dose was obtained from one production cycle [33]. Another recent study of CAR-modified NK-92 suggested the integration of ‘maintenance culture’ that required continuous culture of thawed cells for up to 3 months for investigational medicinal product generation [48]. Such limitations were likely caused by a long recovery period after thawing of frozen NK-92 cells [48] and a relatively slow doubling time (32–50 h) [33, 49]. In this study, we found that NK101 required only two days recovering normal growth profile after cryopreservation, eliminating the need for maintenance culture. Moreover, NK101 cells doubled every 21.9 h while NK-92 cells did every 35.6 h, analogous to a prior study [11], in our culture system. This means that about 1.4 × 10^11^ cells can be produced from 6.25 × 10^6^ cells over 16 days of culture. Assuming that patients in the highest dose group required about 1 × 10^10^ cells/dose, one production cycle could yield about 140 doses. It is also worth highlighting that NK101 express a higher level of high affinity IL-2 receptor, CD25, thus requiring smaller amount exogenous IL-2 for expansion compared to NK-92. Although the optimal scale-up process to maintain the current doubling time of NK101 remains to be developed, the utilization of NK101 over NK-92 for adoptive immunotherapy would bring significant advantages in terms of clinical scale manufacturing.

## Conclusions

Our study presents a newly established NK cell line, NK101, derived from a patient with NK/T cell- lymphoma. NK101 possesses unique phenotypic, genetic and functional features: (i) CD56^dim^CD62L^+^ pro-inflammatory NK cells bearing (ii) molecular signatures associated with immunostimulatory functions that deliver strong antitumor efficacy in immunocompetent hosts and (iii) outstanding potential for large scale manufacturing. Importantly, NK101 is the first NK cell line to exhibit superior in vivo antitumor efficacy compared to NK-92, potentially via the activation of systemic antitumor immunity. Since the clinical success of NK-92-based immunotherapy has been hampered by its limited efficacy and single-patient batch scale production, NK101 may serve as an alternative platform with improved efficacy and superior scalability. Our study raises the need for further explore therapeutic potential of NK101 for future clinical application.

## Additional Files

Additional file 1: **Figure S1.**Histology of the primary biopsy specimens. Biopsied samples from brain lesion were stained with hematoxylin and eosin (left panel) or anti-CD56 antibody (middle panel). Sections of the same samples were also subjected to *in situ* hybridization staining for EBER (right panel). All images were shown at original magnification 400x. **Figure S2.** Analysis of a latent gene and a lytic protein expression of EBV in NK101 cell line. a PCR analysis of *EBNA-2* was performed with gDNAs isolated from B95-8, NK-92, KHYG-1, and NK101 cells. *GAPDH* was used as a loading control. **b** Indicated cells were incubated in the absence or presence of sodium butylate (NaB) and PMA as described in *Online* Additional file 2. Cell lysates were probed with an anti-BZLF1 or anti-β-actin antibody. **Figure S3.** Comparative analysis of cytolytic activities of NK101 and NK-92 against murine tumor cell lines. Indicated murine cell lines were selected as target cells. Indicated cell lines were co-cultured with NK101 (black bar) and NK-92 (white bar) at an effector-to-target ratio of 4:1 for 24 hours. The percentages of apoptotic tumor cells were quantified by Annexin-V and fixable viability dye staining via flow cytometry. Percentage of specific lysis was calculated by the formula described in *Online* Additional file 2. **p<0.01. **Figure S4.** Cytolysis-related gene expression by NK101 and NK-92. GSEA between NK101 and NK-92 was performed in terms of ‘cytolysis’, and the enrichment plot was depicted with NES, *p*-value, and FDR. Expression levels of core genes were illustrated as the heatmap graph. A white-yellow-red color scale indicates expression level transformed as Log2(FPKM+1). **Figure S5.** Effects of conditioned medium derived from NK-92 or NK101 on human peripheral blood mononuclear cells (PBMCs). Human PBMCs labeled with CellTrace Violet (CTV) were cultured under indicated conditions for 5 days. Total cells were stained using the Live/Dead Fixable Viability Dye and fluorochrome-conjugated antibodies specific to CD3, CD4, and CD8. Representative histograms for CTV in the gate of whole PBMCs and CD3- cells are displayed. The results are representative of 2 independent experiments from a single donor in triplicate. SFM, serum-free medium; CM, conditioned medium. **Figure S6.** Pro-/anti-inflammatory gene expression profiles of NK101 and NK-92. Gene expression profiles of cultured NK101 and NK-92 cells were examined by real-time RT-PCR. RNA expression levels for all indicated genes were normalized with a reference gene, *RPL13A*, and calculated based on the 2-ddCt methods as described in *Online* Additional file 2. Relative RNA levels were calculated as follows: 2-ddCt (NK101 or NK-92 RNA level) /2-ddCt (average NK-92 RNA level). Data represent mean ± SD of triplicate wells from 2 independent experiments. **p<0.01; n.s., not significant. **Figure S7.** Expression profiles of cytokines/chemokines from lysates or supernatants of NK101 and NK-92. NK101 or NK-92 cells were cultured under serum-free condition for 72 hours. Both cellular lysates (a) and cultured supernatants (b) were subjected to Proteome Profiler Human Cytokine Array Kit. Differentially expressed cytokines/chemokines are highlighted in boxes. Out of 36 cytokines/chemokines quantified in the lysates and supernatants, levels of 7 proteins with significant difference between NK101 and NK-92 are shown as bar graphs. Data represent mean ± SD of duplicate dots from 2 independent experiments. *p<0.05; **p<0.01; n.s., not significant (PDF 8123 kb)

Additional file 2: Supplementary Methods (DOCX 33 kb)

Additional file 3: **Table S1.** Summary of adventitious agents screening in NK101 cell line (XLSX 11 kb)

Additional file 4: **Table S2.** Summary of gene expression profiles of NK101 and NK-92 by RNA-sequencing. (XLSX 2920 kb) (XLSX 2920 kb)

## Abbreviations

ACT: adoptive cell transfer
BLI: bioluminescence imaging
CAR: chimeric antigen receptor
CD: cluster of differentiation
CFSE: carboxyfluorescein diacetate succinimidyl ester
CHOP-L: L-asparaginase, cyclophosphamide, vincristine, doxorubicin and dexamethasone
CM: conditioned medium
CR: complete remission
CRS: cytokine release syndrome
CTV: CellTrace Violet
DEG: differentially expressed gene
EBV: Epstein-Barr virus
EC_50_: half maximal effective concentration
EGFP: enhanced green fluorescent protein
ELISA: enzyme-linked immunosorbent assay
ELISPOT: enzyme-linked ImmunoSpot
FasL: Fas ligand
FDR: false discovery rate
fLuc: firefly luciferase
GM-CSF: granulocyte-macrophage colony-stimulating factor
GSEA: gene set enrichment analysis
IFN-γ: interferon gamma
IL: interleukin
IP-10: interferon gamma-induced protein 10
KIR: killer cell immunoglobulin-like receptor
LGL: large granular lymphocyte
MCP-1: monocyte chemoattractant protein 1
MHC: major histocompatibility complex
MIP-1b: macrophage inflammatory protein 1 beta
NES: normalized enrichment score
NK: natural killer
PBMC: peripheral blood mononuclear cell
PBS: phosphate buffered saline
PDL: population doubling level
PMA: phorbol myristate acetate
PTI: post tumor injections
SFCs: spot-forming cells
TCR: T cell receptor
TNF-α: tumor necrosis factor alpha
