# Supplementary material for: Discovery of a novel natural killer cell line with distinct immunostimulatory and proliferative potential as an alternative platform for cancer immunotherapy
Source: J Immunother Cancer. 2019 May 24;7:138. doi: 10.1186/s40425-019-0612-2 (PMC6534912; doi:10.1186/s40425-019-0612-2)
Supplement: Supplementary file 1 — Figure S1.Histology of the primary biopsy specimens. Biopsied samples from brain lesion were stained with hematoxylin and eosin (left panel) or anti-CD56 antibody (middle panel). Sections of the same samples were also subjected to in situ hybridization staining for EBER (right panel). All images were shown at original magnification 400x. Figure S2. Analysis of a latent gene and a lytic protein expression of EBV in NK101 cell line. a PCR analysis of EBNA-2 was performed with gDNAs isolated from B95-8, NK-92, KHYG-1, and NK101 cells. GAPDH was used as a loading control. b Indicated cells were incubated in the absence or presence of sodium butylate (NaB) and PMA as described in Online Additional file 2. Cell lysates were probed with an anti-BZLF1 or anti-β-actin antibody. Figure S3. Comparative analysis of cytolytic activities of NK101 and NK-92 against murine tumor cell lines. Indicated murine cell lines were selected as target cells. Indicated cell lines were co-cultured with NK101 (black bar) and NK-92 (white bar) at an effector-to-target ratio of 4:1 for 24 hours. The percentages of apoptotic tumor cells were quantified by Annexin-V and fixable viability dye staining via flow cytometry. Percentage of specific lysis was calculated by the formula described in Online Additional file 2. **p<0.01. Figure S4. Cytolysis-related gene expression by NK101 and NK-92. GSEA between NK101 and NK-92 was performed in terms of ‘cytolysis’, and the enrichment plot was depicted with NES, p-value, and FDR. Expression levels of core genes were illustrated as the heatmap graph. A white-yellow-red color scale indicates expression level transformed as Log2(FPKM+1). Figure S5. Effects of conditioned medium derived from NK-92 or NK101 on human peripheral blood mononuclear cells (PBMCs). Human PBMCs labeled with CellTrace Violet (CTV) were cultured under indicated conditions for 5 days. Total cells were stained using the Live/Dead Fixable Viability Dye and fluorochrome-conjugated antibodies [file 40425_2019_612_MOESM1_ESM.pdf]

# Supplement Figure 1

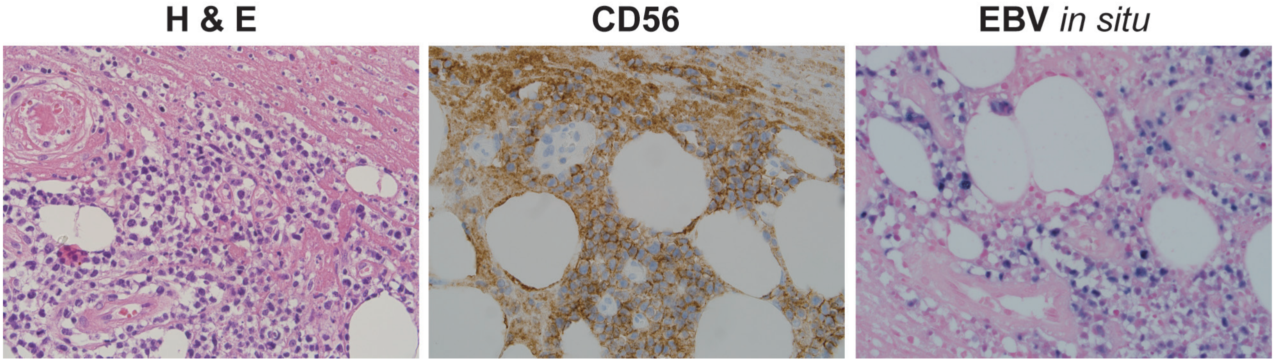


**Supplementary figure 1. Histology of the primary biopsy specimens.** Biopsied samples from brain lesion were stained with hematoxylin and eosin (left panel) or anti-CD56 antibody (middle panel). Sections of the same samples were also subjected to *in situ* hybridization staining for EBER (right panel). All images were shown at original magnification 400x.

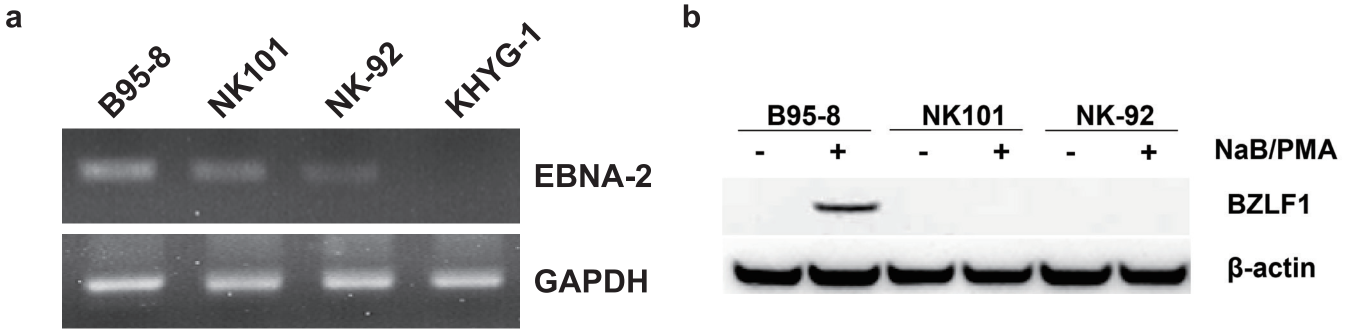


**Supplementary figure 2. Analysis of a latent gene and a lytic protein expression of EBV in NK101 cell line. a** PCR analysis of *EBNA-2* was performed with gDNAs isolated from B95-8, NK-92, KHYG-1, and NK101 cells. *GAPDH* was used as a loading control. **b** Indicated cells were incubated in the absence or presence of sodium butylate (NaB) and PMA as described in *Online Supplementary Methods*. Cell lysates were probed with an anti-BZLF1 or anti-β-actin antibody.

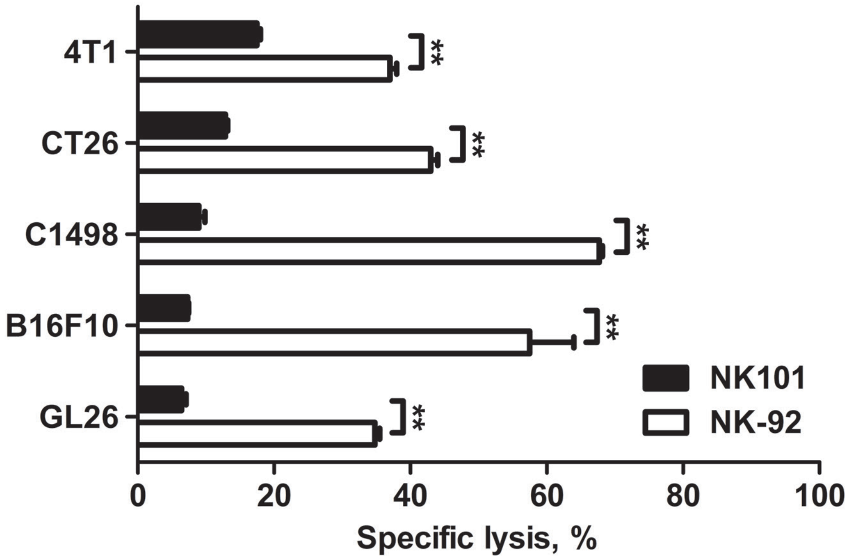


**Supplementary figure 3. Comparative analysis of cytolytic activities of NK101 and NK-92 against murine tumor cell lines.** Indicated murine cell lines were selected as target cells. Indicated cell lines were co-cultured with NK101 (black bar) and NK-92 (white bar) at an effector-to-target ratio of 4:1 for 24 hours. The percentages of apoptotic tumor cells were quantified by Annexin-V and fixable viability dye staining via flow cytometry. Percentage of specific lysis was calculated by the formula described in *Online Supplementary Methods*. **$p$<0.01.

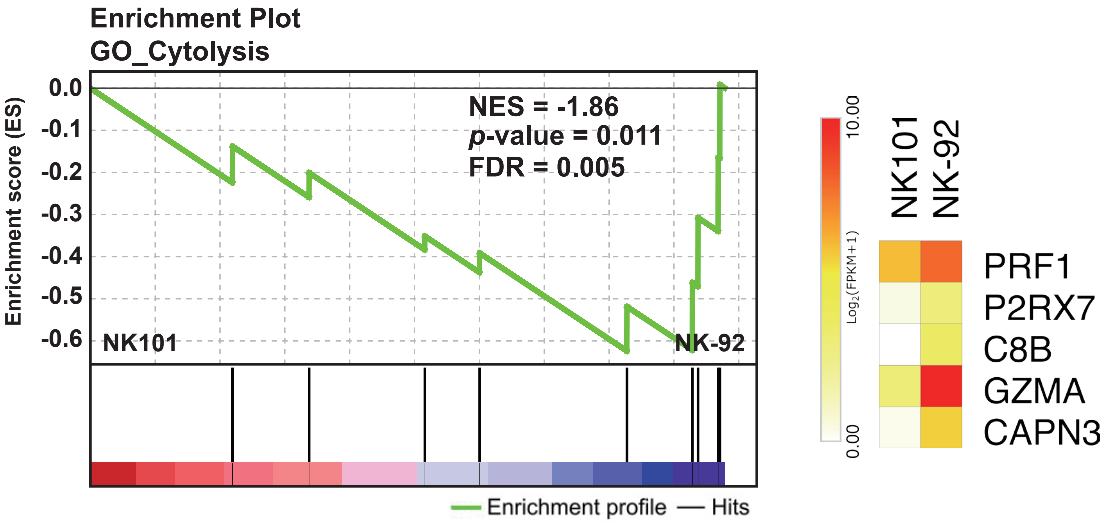


**Supplementary figure 4. Cytolysis-related gene expression by NK101 and NK-92.** GSEA between NK101 and NK-92 was performed in terms of 'cytolysis', and the enrichment plot was depicted with NES, *p*-value, and FDR. Expression levels of core genes were illustrated as the heatmap graph. A white-yellow-red color scale indicates expression level transformed as $Log_2$(FPKM+1).

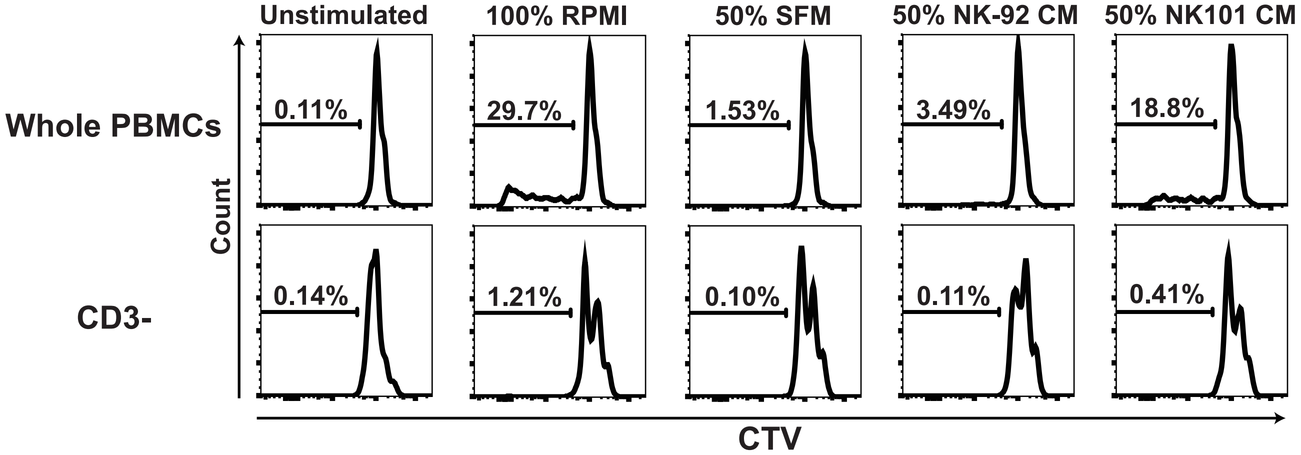


**Supplementary figure 5. Effects of conditioned medium derived from NK-92 or NK101 on human peripheral blood mononuclear cells (PBMCs).** Human PBMCs labeled with CellTrace Violet (CTV) were cultured under indicated conditions for 5 days. Total cells were stained using the Live/Dead Fixable Viability Dye and fluorochrome-conjugated antibodies specific to CD3, CD4, and CD8. Representative histograms for CTV in the gate of whole PBMCs and CD3- cells are displayed. The results are representative of 2 independent experiments from a single donor in triplicate. SFM, serum-free medium; CM, conditioned medium.

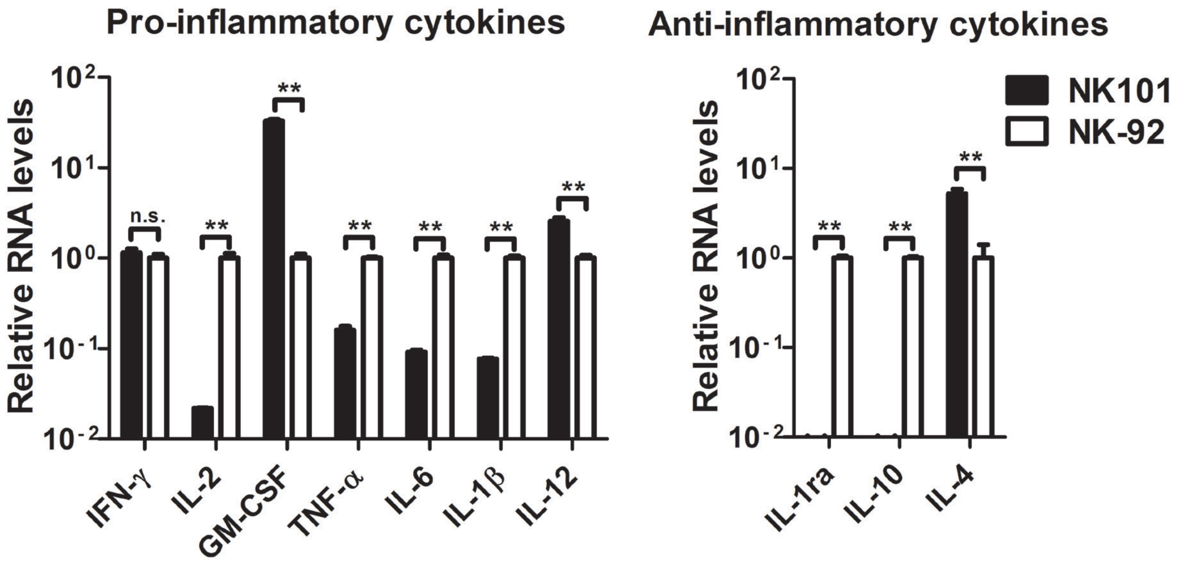


**Supplementary figure 6. Pro-/anti-inflammatory gene expression profiles of NK101 and NK-92.** Gene expression profiles of cultured NK101 and NK-92 cells were examined by real-time RT-PCR. RNA expression levels for all indicated genes were normalized with a reference gene, *RPL13A*, and calculated based on the $2^{-ddCt}$ methods as described in *Online Supplementary Methods*. Relative RNA levels were calculated as follows: $2^{-ddCt}$ (NK101 or NK-92 RNA level) /$2^{-ddCt}$ (average NK-92 RNA level). Data represent mean ± SD of triplicate wells from 2 independent experiments. **$p$<0.01; n.s., not significant.

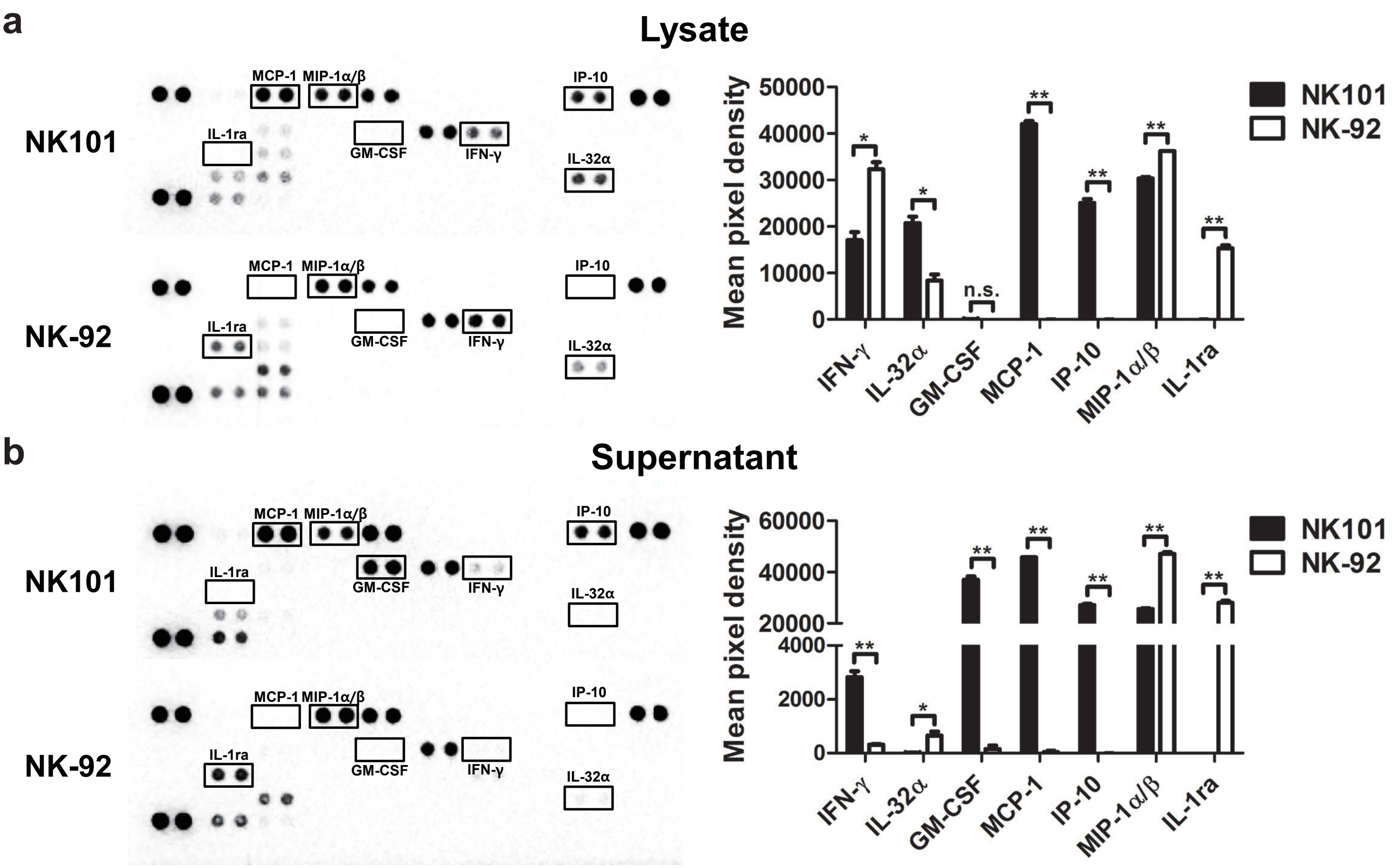


**Supplementary figure 7. Expression profiles of cytokines/chemokines from lysates or supernatants of NK101 and NK-92.** NK101 or NK-92 cells were cultured under serum-free condition for 72 hours. Both cellular lysates (a) and cultured supernatants (b) were subjected to Proteome Profiler Human Cytokine Array Kit. Differentially expressed cytokines/chemokines are highlighted in boxes. Out of 36 cytokines/chemokines quantified in the lysates and supernatants, levels of 7 proteins with significant difference between NK101 and NK-92 are shown as bar graphs. Data represent mean ± SD of duplicate dots from 2 independent experiments. *$p$<0.05; **$p$<0.01; n.s., not significant.
