# Supplementary material for: Discovery of a novel natural killer cell line with distinct immunostimulatory and proliferative potential as an alternative platform for cancer immunotherapy
Source: J Immunother Cancer. 2019 May 24;7:138. doi: 10.1186/s40425-019-0612-2 (PMC6534912; doi:10.1186/s40425-019-0612-2)
Supplement: Supplementary file 2 — Supplementary Methods (DOCX 33 kb) [file 40425_2019_612_MOESM2_ESM.docx]

***Supplementary Methods***

*Cell culture*

OVCAR3, B95-8, THP-1, A549, NCI-H460, SK-BR3, MDA-MB-231, K562, A2780, KG-1, 4T1, CT26 and C1498 cell lines were maintained in RPMI-1640 medium (Hyclone, Logan, UT, USA) with 10% fetal bovine serum (FBS, Hyclone) and 1% penicillin/streptomycin (Gibco-Life Technologies, Rockville, MD, USA), while CaOV3, B16F10, and GL26 cell lines were maintained in Dulbecco’s Modified Eagle’s Medium (DMEM, Hyclone) with 10% FBS and 1% penicillin/streptomycin. All of the above-mentioned cell lines were purchased from Korean Cell Line Bank (KCLB, Seoul, Korea). NK-92 cell line was cultured in Stem Cell Growth Medium (SCGM, CellGenix, Freiburg, Germany) supplemented with 20% FBS, 1% penicillin/streptomycin, and 500IU/ml recombinant human interleukin-2 (rhIL-2, Chiron Corp., San Francisco, CA, USA), and HCT116 cell line was maintained in RPMI-1640 medium with 10% FBS and 1% penicillin/streptomycin. NK-92 and HCT116 cell lines were purchased from the American Type Culture Collection (ATCC, Manassas, VA, USA). All cell lines were incubated at 37℃ with 5% CO_2_, humidified atmosphere.

*Establishment of NK101 cell line*

Mononuclear blood cells were isolated from the lesion by density gradient centrifugation, stimulated with human NK Cell Activation/Expansion kit (Miltenyi Biotec, San Diego, CA, USA) according to the manufacturer’s instructions and cultured in SCGM supplemented with 20% FBS, 1% penicillin/streptomycin, and 500IU/ml of rhIL-2 at 37℃ with 5% CO_2_ in humidified atmosphere. Cells retaining stable growth were sorted into single cells expressing CD56 using MoFlo XDP cell sorter (Beckman Coulter, Brea, CA, USA). A clone displaying exponential growth profile was selected, expanded and designated as NK101. Cell growth was evaluated by population doubling level (PDL). Cumulative PDL was calculated as follows: n = 3.32 (log X – log Y) + Z; n = cumulative PDL, X = harvested cell number, Y = seeded cell number, Z = PDL at previous time point.

*Morphology and immunofluorescence microscopic analysis*

Smears of NK101 cells were prepared by cytocentrifugation at 1000rpm for 3 minutes, stained with Wright-Giemsa solution (Sigma-Aldrich, St. Louis, MO, USA), and visualized by light microscopy. For the immunofluorescence microscopic analysis of perforin and granzyme B, NK101 cells were washed twice with phosphate buffered saline (PBS, Hyclone) and fixed with 4% paraformaldehyde (Affymetrix, Santa Clara, CA, USA). After permeabilization with 4% FBS in 0.1% triton X-100 (Sigma-Aldrich) followed by blocking with 10% normal goat serum (Thermo Fisher Scientific, San Jose, CA, USA), the cells were stained with Alexa Fluor-488-conjugated anti-perforin antibody (Biolegend, San Diego, CA, USA, clone dG9) and Alexa Fluor-647-conjugated anti-granzyme B antibody (Biolegend, clone GB11) diluted in DAKO antibody diluent (DAKO, Glostrup, Denmark) at 1:100 for overnight at 4℃. After repeated washing step, cells were mounted on slide glass with Fluoroshield with DAPI (Immunobioscience, Everett, WA, USA). Images were obtained by using a Leica TCS SP5 II confocal microscopy (Leica Microsystems, Bensheim, Germany) and analyzed with LAS X software (Leica Microsystems).

*Immunophenotypic analysis*

Human TruStain FcX (Biolegend) treated NK101 cells were stained with phycoerythrin (PE)-conjugated anti-CD2 (clone RPA-2.10), -CD3 (clone UCHT1), -CD7 (clone CD7-6B7), -CD11a (clone HI111), -CD14 (clone HCD14), -CD16 (clone B73.1), -CD18 (clone 1B4/CD18), -CD20 (clone 2H7), -CD25 (clone BC96), -CD56 (clone HCD56), -CD85j (clone GHI/75), -CD94 (clone DX22), -CD107a (clone H4A3), -CD122 (clone TU27), -CD132 (clone TUGh4), -TCRαβ (clone IP26), -TCRγδ (clone B1), -NKp30 (clone P30-15), -NKp44 (clone P44-8), -NKp46 (clone 9E2), -NKG2D (clone 1D11), -NKG2A (clone REA110), -KIR2DL1/S1/S3/S5 (clone HP-MA4), -KIR2DL2/DL3 (clone DX27), -ICAM-1 (clone HA58), -perforin (clone dG9), -Granzyme B (clone GB11), -FasL (clone NOK-1), -TRAIL (clone RIK-2), -DNAM-1 (clone 11A8), -CCR1 (clone 5F10B29), -CCR4 (clone L291H4), -CCR5 (clone HEK/1/85a), -CCR6 (clone G034E3), -CCR7 (clone G043H7), -CCR9 (clone L053E8), -CXCR1 (clone 8F1/CXCR1), -CXCR3 (clone G025H7), -CXCR4 (clone 12G5), -CXCR5 (clone J252D4), or -CXCR6 (clone K041E5) antibodies, all of which were purchased from Biolegend, in combination with LIVE/DEAD Fixable Near-IR dye (Molecular Probe, Eugene, OR, USA).

For the comparison of CD56 and CD62L expression level on primary NK cells derived from human peripheral blood mononuclear cells (PBMCs), NK-92 and NK101 cell lines, cells were stained with fluorescein (FITC)-conjugated anti-CD19 (Biolegend, clone HIB19), Brilliant Violet 421 (BV421)-conjugated anti-CD3 (Biolegend, clone SK7), allophycocyanin (APC)-conjugated anti-CD62L (Biolegend, clone DREG-56) and PE-conjugated anti-CD56 antibodies in combination with LIVE/DEAD Fixable Aqua dye (Molecular Probe) after Fc blocker treatment. Data were acquired by BD LSRFortessa flow cytometry (BD, San Jose, CA, USA) and analyzed with FlowJo software (TreeStar, Ashland, OR, USA).

*Cytotoxicity Assay*

Target tumor cell lines were labeled with 5μM carboxyfluorescein diacetate succinimidyl ester (CFSE, Invitrogen, Carlsbad, CA, USA) for 15 minutes at 37℃ with 5% CO_2_ in air. Labeled targets were seeded into 24-well plate at a density of 3x10^5^cells/well, followed by NK-92 or NK101 cell seeding as effector dependent on effector-to-target (E:T) ratio at 4:1 (in case of K562, from 1:1 to 10:1). For the neutralizing antibody assay, 10ng/ml of purified anti-NKG2D (Biolegend, clone 1D11), -NKp30 (Biolegend, clone P30-15), -NKp46 (Biolegend, clone 9E2), -DNAM-1 (Biolegend, clone 11A8), -ICAM-1 (Biolegend, clone HCD54), or isotype-matched antibody (Biolegend, clone MOPC-21) was added at the beginning of co-culture with NK101 and CFSE labeled K562 and THP-1 cells under 4:1 E:T ratio. Percentages of specific lysis and inhibition were calculated as follows:

% specific lysis =

$$\frac{(\% of spontaneous annexin V^{-}LIVE/DEAD dye^{-}) -(\% of sample annexin V^{-}LIVE/DEAD dye^{-})}{(\% of spontaneous annexin V^{-}LIVE/DEAD dye^{-})} \times100\%$$

% of inhibition =

$$\frac{\% specific lysis of isotype control-\% specific lysis of neutralizing antibody treatment group}{\% specific lysis of isotype control}$$

Total cells were harvested after 24 hours incubation, and then stained with annexin V-conjugated with APC (Biolegend) and LIVE/DEAD Fixable Near-IR dye, according to the manufacturer’s instructions. Data were acquired by BD LSRFortessa flow cytometry and analyzed with FlowJo software.

*Preparation of conditioned medium (CM)*

Culture expanded NK101 or NK-92 cells were harvested and centrifuged at 15000 g for 10 minutes at 4℃. Supernatants were removed and cells were resuspended in serum-free SCGM media (CellGenix) at a concentration of 1x10^6^cells/ml. 2 ml suspension was seeded into each well of 6-well plate and incubated at 37℃ with 5% CO_2_ in humidified atmosphere. After 72 hours, the medium were collected and centrifuged at 15000g for 15 minutes at 4℃. Supernatants were harvested and stored at -80℃ for further experiments.

*Human PBMC proliferation/activation assay*

Peripheral blood was obtained with the informed consent of a healthy volunteer and ethical approval by the Institutional Review of Board of the Catholic University of Korea. PBMCs were isolated by density gradient centrifugation, maintained in RPMI-1640 medium (Hyclone) with 10% FBS (Hyclone) and 1% penicillin/streptomycin (Gibco-Life Technologies) for 6 hours at 37℃ with 5% CO_2_, humidified atmosphere for stabilization. Nextly, PBMCs were harvested and labeled with CellTrace Violet Cell Proliferation Kit (CTV, ThermoFisher Scientific) according to the manufacturer’s guideline. Prior to the cell seeding, 48-well plates were coated with 5μg/ml of purified anti-CD3 antibody (BD, clone SP34-2) diluted in PBS (Hyclone) for 2 hours at 37℃ followed by three washing steps with fresh PBS. 4x10^5^ cells of human PBMCs resuspended in 250μl RPMI1640 medium (Hyclone) supplemented 10% FBS (Hyclone) were seeded into each well of the 48-well plate, and the same volume of NK101 or NK-92 conditioned medium (CM) was added, generating 50% CM. Unstimulated, serum-free SCGM (CellGenix) added (50% SFM), and 10% RPMI1640 added wells were included as controls. Total cells were harvested after 5 days and washed with PBS, followed by blocking with Human TruStain FcX (Biolegend).

For the assessment of PBMC proliferation, whole PBMCs were stained with FITC-conjugated anti-CD3 (Biolegend, clone HIT3a), PE-conjugated anti-CD4 (Biolegend, clone A161A1), APC-conjugated anti-CD8 (Biolegend, clone SK1) antibodies combination with LIVE/DEAD Fixable Near-IR Dye (Molecular Probe).

For the assessment of PBMC activation, whole PBMCs were stained with FITC-conjugated anti-CD3 (Biolegend, clone HIT3a), BV605-conjugated anti-CD25 (Biolegend, clone BC96), PE-conjugated anti-CD69 (BD, clone FN50) antibodies combination with LIVE/DEAD Fixable Near-IR Dye (Molecular Probe).

Data were acquired by BD LSRFortessa flow cytometry (BD) and analyzed with FlowJo software (TreeStar).

*Cytokine measurements*

Rested NK101 cells (3x10^5^) were incubated with 500IU/ml of IL-2 or 10ng/ml of IL-12 (PeproTech, London, UK), IL-15 (PeproTech), IL-18 (PeproTech), or IL-21 (PeproTech) for 24 hours. Supernatants were collected and stored at -20℃ for subsequent analysis. The quantitation of IFN-γ was performed by ELISA kit (R&D Systems, Minneapolis, MN, USA) under manufacture’s instruction with the culture supernatants. Concentrations of multiple cytokines and chemokines were measured from the culture supernatants of rested or co-cultured NK101 with K562 or THP-1 cells at 4:1 E:T ratio by multiplex immunoassay (Luminex) (R&D Systems) according to the manufacturer’s instructions.

For the quantification of pro- or anti-inflammatory cytokine levels secreted by NK-92 and NK101, CM derived from each cell line was examined to measure the levels of IFN-γ, TNF-α, GM-CSF, IL-2, IL-6, IL-1β, IL-12, IL-1ra, IL-10, or IL-4 using respective ELISA kit (R&D Systems).

For the comparative analysis of various cytokines and chemokines produced by NK-92 and NK101, cells (1x10^6^) were cultured under serum-free SCGM (CellGenix) for 72 hours. The relative levels of multiple cytokines and chemokines in cellular lysates and supernatants were measured using Human Cytokine Array (R&D Systems) according to the manufacturer’s instructions. The spot intensities were analyzed using the ImageJ software v1.5.1j8 (NIH, Bethesda, MD, USA).

*Cell proliferation assay*

Cultured NK101 cells were seeded at 2x10^4^cells/well in 96-well plate, treated with various cytokines, and maintained at 37℃ with 5% CO_2_ in humidified atmosphere for 3 days. Cell proliferation was evaluated by MTS assay using the CellTiter 96 Aqueous assay kit (Promega, Madison, WI, USA) following manufacturer’s instruction. Absorbance at 490nm was measured by ELISA plate reader (Molecular device, San Jose, CA, USA). Fold expansion was calculated as follows:

Fold expansion = $\frac{\text{O.D.}\text{sample}-\text{O.D.}\text{media only}}{\text{O.D.}\text{no cytokine}-\text{O.D.}\text{media only}}$

*Polymerase chain reaction*

gDNA of B95-8, NK101, NK-92, and KHYG-1 was prepared by gDNA preparation kit (Macherey-Nagel, Duren, Germany) according to the manufacturer’s instructions. PCR was performed with EBNA-2 or GAPDH specific primers. Primer information is as follows;

EBNA-2 (forward): 5’-AGGGATGCCTGGACACAAGA-3’

EBNA-2 (reverse): 5’-GCCTCGGTTGTGACAGAG-3’

GAPDH (forward): 5’-ACCACAGTCCATGCCATCAC-3’

GAPDH (reverse): 5’-TCCACCACCCTGTTGCTGTA-3’

*Western blotting*

B95-8, NK101 and NK-92 cells were stimulated with 2.5mM of sodium butylate (Sigma-Aldrich) and 20ng/ml of PMA (Sigma-Aldrich) or left unstimulated for 48 hours. Harvested cells were washed with PBS, and then lysed with PRO-PREP protein extraction solution (Intron, Seongnam, Korea) on ice for 20 minutes. After centrifugation at 13000rpm for 10 minutes at 4℃, whole cell lysates were collected and protein concentration was quantified using Pierce BCA Protein Assay kit (Thermo Fisher Scientific). Lysates (100μg) were subjected to electrophoresis and transferred into 0.2μm nitrocellulose membranes (Invitrogen). After an hour of blocking procedure with 5% (w/v) skimmed milk (BD) in Tris-buffered saline with 2% Tween-20 (Sigma-Aldrich), anti-EBV ZEBRA antibody (Santa Cruz, Dallas, TX, USA, 1:100) or anti-β-actin antibody (Santa Cruz, 1:3000) were added to the membranes and maintained for 3 hours later at room temperature. Nextly, the membranes repeatedly washed with PBS and were further incubated with goat anti-mouse IgG-HRP antibody (Santa Cruz, 1:1000) for 3 hours at room temperature. After PBS washing step, bands in the membranes were visualized by ChemiDoc (BioRad, Hercules, CA, USA).

*Real-time RT-PCR*

Culture expanded NK101 or NK-92 cells were harvested and 5x10^6^ cells were subjected to RNA isolation. Total RNA was isolated using *AccuPrep* Universal RNA Extraction kit (Bioneer, South Korea) and cDNA was synthesized from the 1μg total RNA using *AccuPower^®^* Rocketscript^TM^ Cycle RT Premix (Bioneer) according to the manufacturer’s instruction. A mixture for RT-PCR reaction was prepared with 1μl of cDNA, 5μl of primer sets, 15μl of deionized sterile water, and 25μl of *AccuPower* 2X GreenStar Master Mix (Bioneer). Real-time RT-PCR was performed on Exicycler 96 Real-Time Quantitative Thermal Block (Bioneer) with cycling condition as follows: 40 cycles at 95℃ for 5 sec, 58℃ for 25 sec, 72℃ for 30 sec.

The primer sequences are the following (5’-3’):

IFN-γ (F: TGGGTTCTCTTGGCTGTTACT, R: TCTGTCACTCTCCTCTTTCCAA),

IL-2 (F: GCAACTCCTGTCTTGCATTG, R: GAGCATCCTGGTGAGTTTGG),

GM-CSF (F: GGGAGCATGTGAATGCCATC, R: CTGTTTCATTCATCTCAGCAGC), TNF-α (F: CTGCACTTTGGAGTGATCGG, R: CAGCTTGAGGGTTTGCTACA),

IL-6 (F: AATAACCACCCCTGACCCAA, R: GCTACATTTGCCGAAGAGCC),

IL-1β (F: AGCAACAAGTGGTGTTCTCC, R: TGGGATCTACACTCTCCAGC),

IL-12 (F: TCCTGGACCACCTCAGTTTG, R: GTTTGTCTGGCCTTCTGGAG),

IL-1ra (F: AGCCTCATGCTCTGTTCTTG, R: TGTCCTGCTTTCTGTTCTCG),

IL-10 (F: GGAGAACCTGAAGACCCTC, R: TGGCTTTGTAGATGCCTTTC),

IL-4 (F: CATGAGAAGGACACTCGCTG, R: CGTACTCTGGTTGGCTTCC).

RPL13A (F: GTGTTTGACGGCATCCCACC, R: TAGGCTTCAGACGCACGACC).

RPL13A gene was utilized as a reference gene to normalize all data calculating ddCt value as follows: ddCt = [Ct(target gene)-Ct(RPL13A)]. Expression level of individual gene was quantified based on the 2^-ddCt^ method.

*Mouse IFN-γ ELISPOT*

Mouse IFN-γ ELISPOT (BD) was performed according to the instructions of manufacturer. Briefly, 2.5x10^5^ cells of mouse splenocytes were stimulated for 24 hours with complete culture medium (negative control), concanavalin A (Sigma-Aldrich, positive control, 5μg/ml), or 4T1 lysates (50μg/ml) prepared by repeated freezing and thawing. IFN-γ positive spots were recorded and counted by AID ELISPOT Reader (AID, Strassberg, Germany). The number of spot-forming cells (SFCs) was adjusted to 1x10^6^ cells of splenocytes.
